# Functionalized bioresorbable biliary stents: materials design, coating engineering, and clinical translation

**DOI:** 10.1039/d6ra06308j

**Published:** 2026-10-19

**Authors:** Bowen Wang, Wei Gong

**Affiliations:** a Department of Pancreatic Disease Diagnosis and Treatment Center, Shandong Provincial Hospital Affiliated to Shandong First Medical University Jinan 250021 Shandong China gongwei75slyy@163.com

## Abstract

Biliary strictures remain a major therapeutic challenge because conventional plastic and self-expandable metal stents are limited by occlusion, repeat intervention, migration, and tumor ingrowth. Bioresorbable biliary stents offer temporary mechanical scaffolding and, when functionalized, can also provide local therapeutic delivery without permanent foreign-body retention. This review focuses on the engineering decisions that determine whether these devices can move beyond descriptive promise toward indication-specific translation. We compare polymeric and emerging metallic biodegradable platforms, distinguish *in vitro*, preclinical *in vivo*, and expected real-bile degradation behavior, and critically evaluate the trade-offs among radial force retention, flexibility, fatigue resistance, radiopacity, drug release, biofilm resistance, manufacturability, and clinical readiness. Current evidence suggests that polydioxanone (PDO)- and copolymer-based designs may be more suitable for benign or post-surgical indications requiring temporary support, whereas malignant obstruction may require longer patency, stronger radial force, and carefully controlled antiproliferative or chemotherapeutic release. However, available human data include selected cohorts and registries but remain limited in comparative strength, so claims regarding long-term efficacy, quality of life, and cost-effectiveness should remain cautious until larger comparative trials are available.

## Abstract 2

Bioresorbable biliary stents combine controlled scaffold degradation, radiographic trackability, and therapeutic coatings for indication-specific clinical translation.

## Graphical abstract

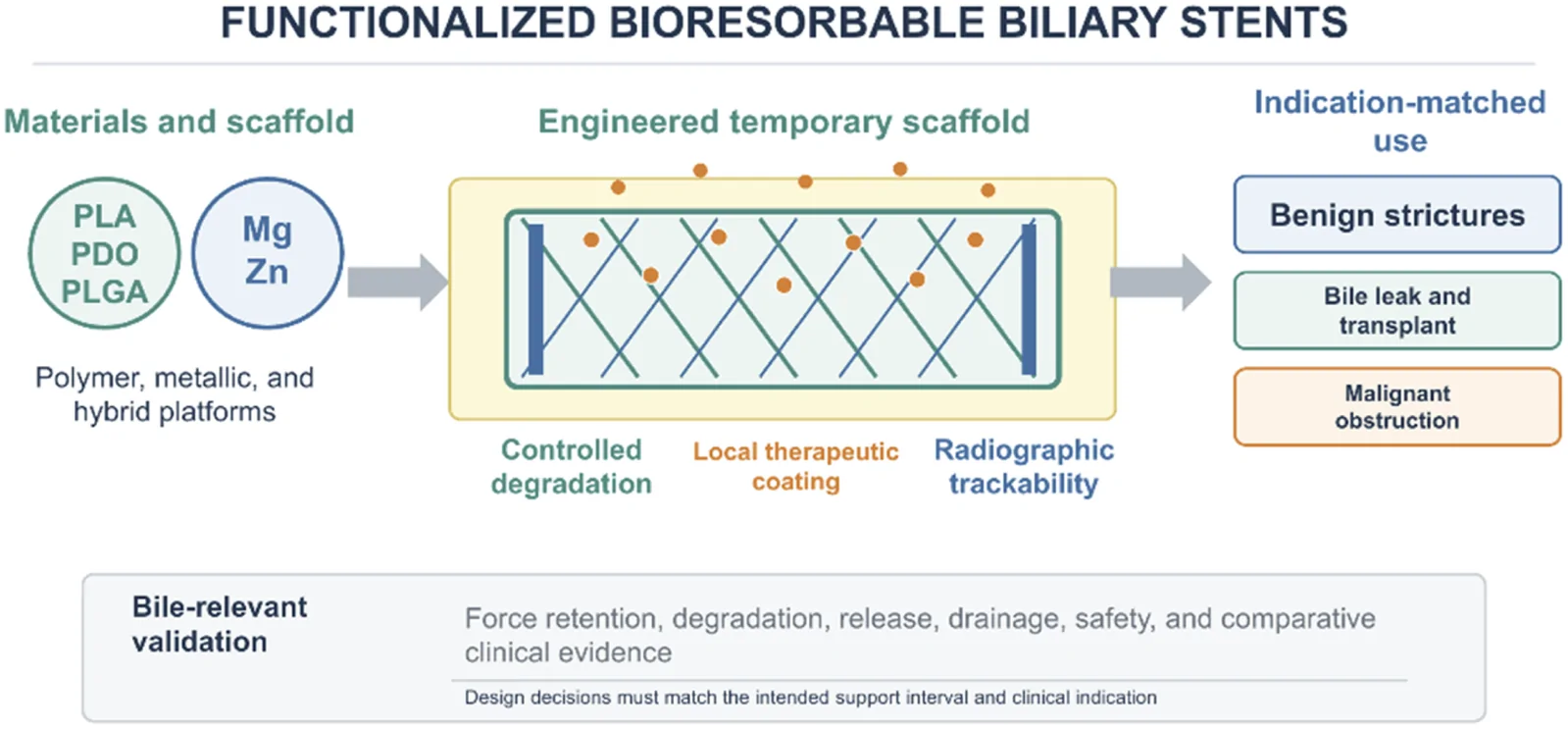

## 1. Introduction

Biliary strictures refer to pathological narrowing of the bile duct lumen and constitute a major clinical problem encountered in hepatobiliary practice. These strictures originate from diverse etiological factors, and their prevalence differs according to the specific underlying pathology. The clinical consequences of biliary strictures can be severe, including obstructive jaundice, cholangitis, liver dysfunction, and eventually liver failure if left untreated.^1^

Current treatment options for biliary strictures include endoscopic stent placement, surgical reconstruction, and percutaneous drainage, each with significant limitations.

Because of its minimally invasive characteristics, endoscopic stent placement has become the primary therapeutic option, but it remains associated with considerable complication and failure rates. Traditional plastic stents have limited patency and may require replacement procedures, thereby increasing both patient morbidity and the overall burden on healthcare resources. The short patency time of plastic stents is primarily due to their relatively small diameter (typically 7–10 French) which predisposes them to obstruction caused by biliary sludge accumulation and debris deposition. Biliary sludge deposition is the most common cause of plastic stent occlusion. In addition to their short patency time, plastic stents have several other limitations. They are relatively rigid and may induce mechanical injury to the bile duct during insertion, particularly in patients with strict or tortuous anatomy. Plastic stents also exhibit a risk of migration, which may subsequently result in complications including cholangitis, pancreatitis, and perforation.^1^

Self-expandable metal stents (SEMS) provide a larger lumen than plastic stents. Uncovered SEMS can be complicated by tumor ingrowth and recurrent obstruction in malignant disease. Tumor ingrowth occurs when cancer cells penetrate through the lattice structure of the stent, leading to stent occlusion and the need for additional interventions. Covered SEMS have been developed to reduce the risk of tumor ingrowth, yet these devices show an increased incidence of stent migration and cholecystitis. Cholecystitis can occur when the stent obstructs the cystic duct opening, preventing bile flow to the gallbladder. Uncovered SEMS can become embedded and difficult to remove, whereas fully covered SEMS (FCSEMS) are designed to permit removal.^1^

These shortcomings have prompted the exploration of bioresorbable biliary stents capable of offering temporary structural support while progressively degrading within the body, potentially avoiding the need for secondary removal procedures. Bioresorbable stents are designed to maintain mechanical integrity for a predetermined period of time to allow for tissue healing, after which the structure gradually breaks down into biocompatible byproducts that are cleared through physiological metabolic processes.^2^

However, the translation of this technology to the biliary tract presents unique challenges due to the complex physiological environment, including exposure to bile acids, enzymatic activity, and mechanical stress from peristalsis. The biliary tract is also subject to significant variations in pressure and flow, which can affect stent performance and degradation kinetics. Functionalized bioresorbable biliary stents incorporate additional therapeutic capabilities through surface coatings and drug delivery systems, aiming to enhance tissue healing and prevent complications such as restenosis, infection, and stone formation. Investigational coating strategies encompass multiple therapeutic compounds, including antiproliferative drugs, anti-inflammatory agents, antibiotics, and growth factors, through controlled and site-specific release profiles. This targeted delivery approach has the potential to improve therapeutic outcomes while simultaneously limiting systemic drug exposure and the risk of treatment-related adverse reactions. Potential benefits and their evidence limitations include:^3^

(1) Avoidance of planned removal procedures: bioresorbable stents are engineered to gradually degrade *in vivo*, potentially avoiding additional endoscopic or surgical procedures required for stent retrieval. Avoiding secondary removal interventions may reduce procedure-related burden, although migration, fragmentation, or recurrent obstruction can still require rescue intervention.^4^

(2) Temporary mechanical support: bioresorbable stents are intended to preserve structural strength for a predetermined period that allows sufficient tissue repair, after which the material progressively breaks down as the bile duct recovers. This design aims to provide temporary luminal support while preventing the long-term retention of a permanent foreign body within the biliary tract.

(3) Localized drug delivery: functionalized bioresorbable stents aim to enable site-specific administration of therapeutic agents directly to the affected biliary region, with the goal of enhancing local therapeutic performance while reducing systemic toxicity. Such localized delivery may facilitate tissue regeneration and lower the risk of complications including restenosis and infection.

(4) Reduced risk of complications: lower rates of migration, tumor ingrowth, and biliary sludge deposition are design objectives rather than established benefits over traditional stents. The gradual degradation of the stent may mitigate chronic foreign body reactions, but fragmentation can introduce additional risks.^2,5^

(5) Patient quality of life: avoiding stent removal procedures may lessen psychological stress, physical discomfort, and interference with normal daily activities. These potential benefits require standardized patient-reported outcome measurements; fewer scheduled procedures alone do not establish improved quality of life.^4^

Despite these potential benefits, several technical and clinical challenges remain before bioresorbable biliary stents can achieve broad clinical implementation. These include the need for accurate regulation of degradation kinetics, the development of materials and structural designs capable of tolerating the chemically aggressive biliary environment, and the need for well-designed clinical studies to verify long-term safety and therapeutic effectiveness.^3^

At the outset, the overall conceptual framework of bioresorbable biliary stents is illustrated in Fig. 1. The schematic integrates the clinical limitations of conventional stenting strategies with engineering solutions based on biodegradable scaffolds, functional surface coatings, radiopaque modification, and translational assessment. Biodegradable describes material breakdown through chemical or biological processes; bioresorbable denotes a device intended to undergo breakdown and subsequent biological clearance. Degradation is not itself proof of resorption. Polymer chain scission, measurable mass loss, loss of radial support, fragmentation, and imaging disappearance are reported as separate endpoints. Patency duration is a time interval; patency rate is the proportion of patent devices or treated patients at a specified time. Evidence is identified as laboratory testing, biliary animal testing, human biliary use, or non-biliary extrapolation. A degradable coating on a persistent metal framework is distinguished from a fully bioresorbable stent. Existing reviews already integrate biodegradable biliary materials, functional coatings, and clinical translation, including those by Song, Li, Yang, Yan, and particularly Tang and colleagues.^2,3,6–8^ The contribution here is a critical synthesis organized around three decisions: which degradation endpoint is relevant to functional drainage, how directly each technology has been tested in the biliary tract, and which indication can justify its mechanical and therapeutic trade-offs. The subsequent sections therefore move from material and mechanical design to surface functionality and clinical translation, with particular attention to the conditions under which each approach appears promising or remains insufficiently validated.

**Fig. 1:**
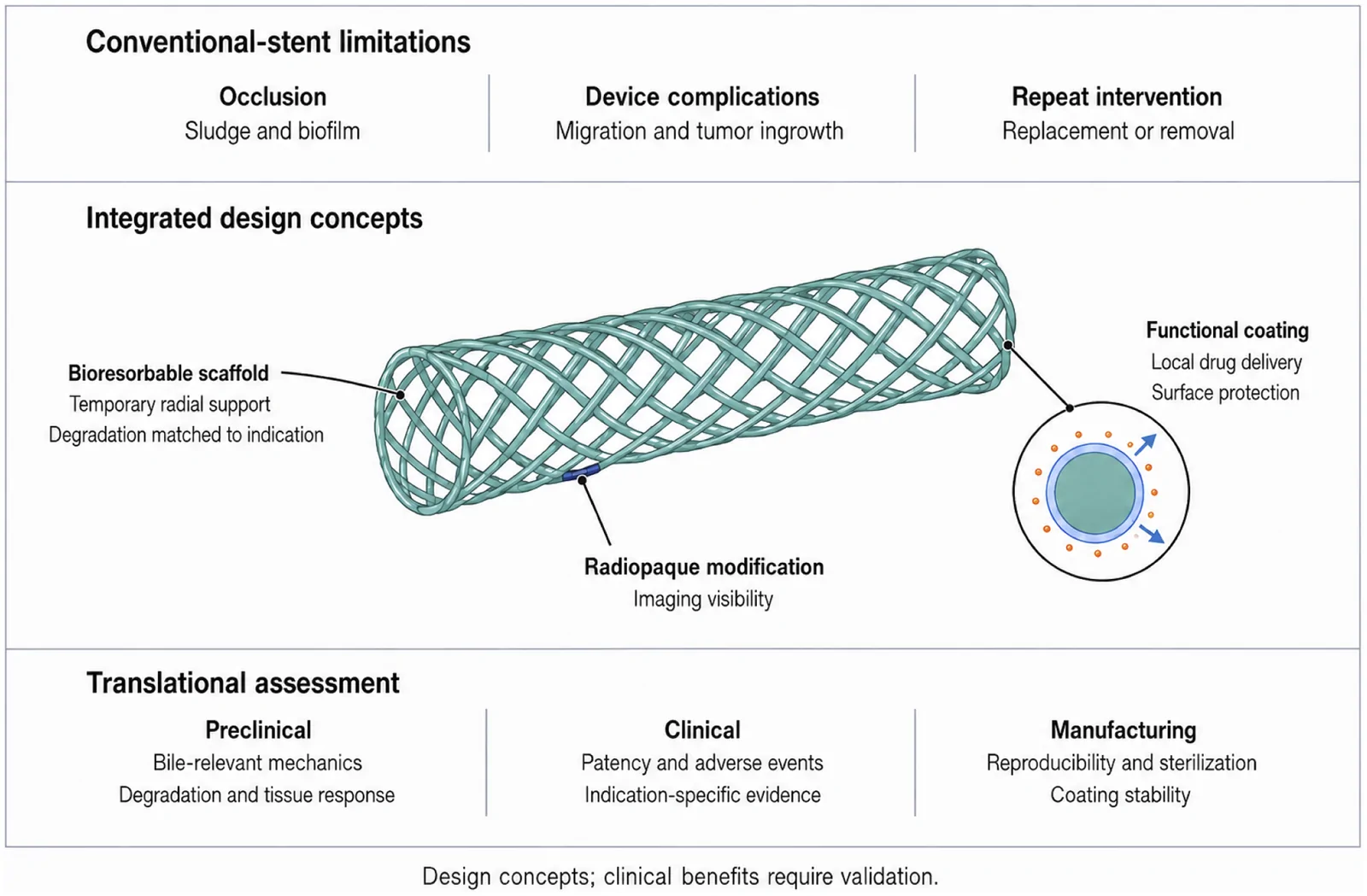
Conceptual framework of functionalized bioresorbable biliary stents. The upper panel depicts conventional-stent limitations; the middle panel combines scaffold, coating, and imaging concepts; the lower panel outlines preclinical, clinical, and manufacturing assessment. These are design concepts rather than established clinical benefits.

This narrative review was informed by targeted searches of PubMed and publisher websites, with DOI and bibliographic cross-checking through Crossref and reference-list screening. Bibliographic and targeted source checks were updated in October 2026 and included foundational studies and relevant publications available through 7 October 2026. Search combinations included biliary stent with biodegradable or bioresorbable, polydioxanone, polylactide, magnesium, zinc, degradation, radiopacity, drug elution, biofilm, benign stricture, bile leak, and transplantation. Primary studies were prioritized for quantitative outcomes; reviews provided context and helped identify earlier reports. Selection emphasized an identifiable device, anatomical setting, test environment, and endpoint. Evidence from other implants was retained only for explicitly identified design concepts. This is a selective narrative synthesis, without a prospectively registered protocol, exhaustive database screening, or meta-analysis; selection bias and incomplete coverage remain possible.

## 2. Materials design for bioresorbable biliary stents

### 2.1 Polymer selection and properties

Choosing suitable biomaterials represents a fundamental requirement in the effective design of bioresorbable biliary stents. The ideal material must possess a combination of properties including sufficient mechanical strength to maintain patency during the healing period, controlled degradation kinetics matching the tissue repair timeline, excellent biocompatibility to minimize inflammatory responses, and processability to allow for manufacturing of complex stent geometries. Currently, the most commonly used polymers for bioresorbable biliary stents include poly(lactic acid) (PLA), poly(glycolic acid) (PGA), polydioxanone (PDO), and their copolymers. The choice of polymer depends on several factors, including the desired degradation timeline, required mechanical properties, intended clinical application, and manufacturing considerations. Each polymer has unique advantages and limitations that need to be carefully considered when designing bioresorbable biliary stents. A clinically useful comparison should therefore move beyond listing polymer properties and ask which material profile fits each biliary scenario. Benign strictures and bile leaks usually require temporary scaffolding during healing, favoring materials with predictable support over a disease-specific healing interval. Malignant obstruction, by contrast, may demand stronger radial force, longer patency, and compatibility with local antitumor therapy. Post-transplant anastomotic strictures require particular caution because local inflammation, infection risk, and the need to avoid repeat intervention must be balanced against the consequences of premature fragmentation. Although most current biliary systems are polymer-based, biodegradable metallic materials, especially zinc-based alloys, are now being examined as alternatives because they may combine radiopacity, mechanical strength, and gradual corrosion-driven resorption.^2,3,9^

#### 2.1.1 PLA

PLA belongs to the family of aliphatic polyesters and is typically synthesized from renewable raw materials, including corn starch and sugarcane. Within the physiological environment, PLA is degraded mainly through hydrolytic cleavage of its ester bonds, generating lactic acid as the primary degradation product. This metabolite subsequently enters the tricarboxylic acid cycle and is ultimately converted into carbon dioxide and water for elimination. The degradation duration of PLA is not fixed and varies depending on factors such as molecular weight, polymer crystallinity, and processing conditions during fabrication.^10^

PLA is present in two stereochemical configurations, poly(l-lactic acid) (PLLA) and poly(d-lactic acid) (PDLA). Among these, PLLA is more frequently employed in medical devices owing to its relatively slow degradation profile. The mechanical behavior of PLA-based materials can be tuned through adjustments in molecular weight, where polymers with higher molecular weights generally demonstrate greater tensile strength and higher elastic modulus.^2,10^

In the porcine study by Zhang *et al.*, mean patency was 25.7 ± 5.6 weeks for PLA-based stents *versus* 11.3 ± 3.4 weeks for PDO (five pigs per analyzed group). Five of 15 initially assigned animals were excluded, including three with migration. Fig. 2 shows structural changes on radiographs; patency is not a measurement of complete resorption.^11^ This duration should be interpreted critically rather than solely as an advantage. Prolonged persistence may be useful for complex strictures or delayed healing, but it may also extend exposure to acidic degradation products, chronic foreign-body irritation, biliary sludge accumulation, and coating instability. Thus, PLA is better viewed as a long-support option for selected indications than as a universally optimal polymer.

**Fig. 2:**
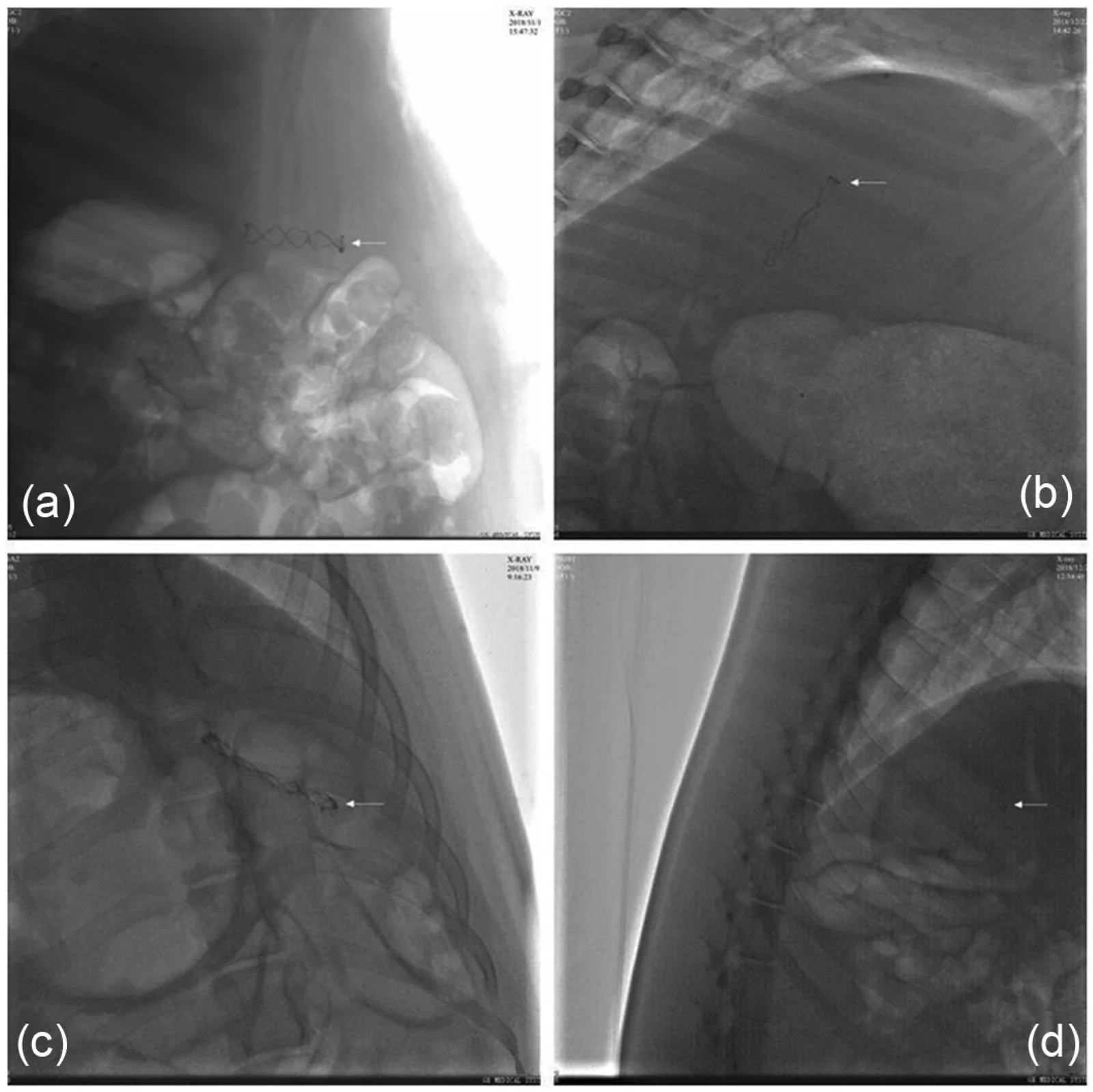
X-ray imaging of the stent degradation process (arrow shows the lead mark) ((a), upper left) normal structure; ((b), upper right) partial structural deformation; ((c), lower left) complete structural deformation; ((d), lower right) chaotic structure. These radiographic appearances do not directly quantify radial force or complete biological resorption.^11^

Compared with PDO and PLGA, the persistence of a PLA device may exceed the desired healing interval in indications that only require short-term scaffolding. PDO offers a flexible framework with device-dependent degradation, whereas PLGA allows degradation to be tuned by changing the lactide : glycolide ratio. For malignant obstruction, the longer persistence of PLA may help maintain patency, but this advantage must be weighed against local inflammation, sludge deposition, and the uncertain ability of PLA-based systems to deliver drugs at therapeutically meaningful concentrations over the full patency period.^2,12^

#### 2.1.2 PGA

PGA is another widely used biodegradable polymer with faster degradation kinetics compared to PLA. PGA degrades in physiological environments mainly through hydrolytic cleavage of its ester bonds, generating glycolic acid as the principal breakdown product. This metabolite is subsequently processed through the tricarboxylic acid cycle and eliminated through normal metabolic pathways. Compared with PLA, PGA generally exhibits a faster degradation profile, which is primarily related to its greater hydrophilic character. The higher affinity for water facilitates deeper water diffusion into the polymer matrix, thereby accelerating hydrolysis of the polymer backbone.^2,10^

Despite this advantage in degradation responsiveness, the rapid breakdown of PGA can lead to an early decline in structural integrity, potentially resulting in insufficient mechanical support during the full period required for tissue recovery in certain clinical situations. In a study evaluating PGA biliary stents in an *in vitro* bile-flow phantom, complete degradation was observed within 6 weeks, which may be too rapid for treating chronic biliary strictures requiring prolonged support. Effects of bile composition depend on the polymer and test environment and cannot be represented by a universal acceleration factor.^5,10^

PGA also has a tendency to crystallize during degradation, which can affect its mechanical properties and degradation kinetics. The crystallization process can lead to increased brittleness and reduced flexibility, which may increase the risk of stent fracture or migration. High initial strength alone does not establish sustained function; force retention and fragmentation should be measured during degradation.^5,10^

#### 2.1.3 PDO

PDO is a biodegradable polyester whose degradation depends on processing and device geometry. PDO offers flexibility, making it suitable for self-expandable stent designs that need to conform to the irregular geometry of bile ducts. Hydrolytic chain cleavage changes its mechanical properties over time; a bulk-polymer resorption range does not establish a stent support interval.^2,5^

In a porcine model, PDO stents retained their structural morphology for nearly 12 weeks, but showed more pronounced fragmentation and promoted biliary stone formation compared with PDO/PLLA composite stents. The principal limitation of PDO is therefore the risk that fragmentation and acidic degradation products may interact with bile constituents to promote sludge or stone formation. Composite designs can partly address this issue by pairing a flexible PDO component with a slower-degrading PLLA core or other reinforcing phase.^5^

#### 2.1.4 Copolymers

Copolymers such as poly(lactic-*co*-glycolic acid) (PLGA) provide the ability to regulate degradation behavior through modification of the lactide to glycolide composition. The mechanical properties of PLGA can also be tailored by adjusting the monomer ratio, although the effect also depends on molecular weight, architecture, and processing. Other copolymers that are considered for degradable device design include poly(lactic acid-*co*-caprolactone) (PLCL) and poly(glycolic acid-*co*-caprolactone) (PGCL). Caprolactone segments can increase flexibility, supporting their consideration for self-expandable stent designs.^2,10,12^

PLCL is a copolymer of lactic acid and caprolactone. Incorporation of caprolactone segments enhances polymer flexibility and toughness, with composition-dependent degradation. PLCL has been shown to have good biocompatibility and mechanical properties, which supports its potential use in biliary stent systems. The discussion of PGCL is a material-design possibility rather than direct evidence of validated biliary-stent performance.^2,13^

One of the main advantages of copolymers is their ability to combine the beneficial properties of different monomers. However, copolymers also have some limitations, including more complex manufacturing processes and potential batch-to-batch variability. The degradation kinetics of copolymers can also be more difficult to predict compared to homopolymers, which can make it challenging to design stents with consistent performance. In blends or block architectures, an additional challenge is potential phase segregation, which may alter both the mechanical behavior and the degradation dynamics of the copolymer material. This phenomenon may result in heterogeneous degradation behavior, potentially causing complications such as stent fragmentation or premature loss of mechanical integrity. Table 1 summarizes and compares the principal characteristics of different bioresorbable polymers used in biliary stent design, using device-specific environments and endpoints rather than unmatched bulk mechanical values.^10^

**Table 1:** Comparison of bioresorbable polymers for biliary stent applications^a^

Platform	Environment and endpoint	Direct observation	Design implication and limitation	Source
PLA	Porcine biliary study; analyzed-group patency and structural change	Mean patency 25.7 ± 5.6 weeks (5 pigs); PDO comparator 11.3 ± 3.4 weeks (5 pigs)	Five of 15 initially assigned animals excluded, including three migrations; patency and filament breakage are not complete resorption	11
PGA	*In vitro* bile-flow phantom; complete device degradation as reported	Approximately 6 weeks	Rapid loss of framework may be unsuitable for prolonged support; not a swine resorption time	5
PDO	Porcine biliary implantation; macroscopic integrity/disappearance	Basic morphology about 12 weeks in one study; absorption observed at 13- and 20 weeks assessments in another	Flexible; fragmentation, stone formation, and obstruction remain device-dependent risks	5,14
PDO sheath/PLLA core	*In vitro* bile-flow phantom; radial-force and structural retention	Retention up to approximately 16 weeks	Composite reinforcement prolongs retention in the tested system; human effectiveness unestablished	5
PLGA 71:29	Human bile *in vitro*; subsequent rat bile-duct implantation	Formulation selection by weight loss; device disappearance at 4–5 weeks in rats	Endpoint and environment must accompany each duration; no generic resorption time assigned	12
PLCL	Porcine biliary implantation of radiopaque devices	CT visibility at 2 months and disappearance by 6 months	Flexible composition and imaging modification; disappearance on CT is not proof of full clearance	13

a Times are observations from individual devices and protocols, not recommended treatment durations. Mechanical support, fragmentation, mass loss, and biological resorption require separate measurements. Bulk tensile properties cannot be compared reliably without matched specimen geometry, hydration, and test conditions.

### 2.2 Mechanical design and degradation kinetics

#### 2.2.1 Mechanical properties and design considerations

The mechanical design of bioresorbable biliary stents must balance multiple competing requirements, including sufficient radial force to maintain patency, flexibility to accommodate anatomical variations and peristaltic movements, and fatigue resistance to withstand repeated mechanical stress over time. Self-expandable designs are particularly attractive for biliary applications as they can be delivered through minimally invasive endoscopic procedures and adapt to the irregular geometry of bile ducts.^2^

Radial force represents a key mechanical parameter governing whether a stent can preserve bile duct patency, but it cannot be evaluated in isolation. Higher outward force may be necessary in malignant obstruction because tumor compression can be substantial, whereas excessive force in benign strictures may aggravate epithelial injury and fibrosis. In the study by Kwon *et al.*, Fig. 3 shows the morphology and sheath-core architecture of the monofilaments, rather than radial-force measurements. Force comparisons require matched device dimensions, compression protocols, and aging conditions.^5^

**Fig. 3:**
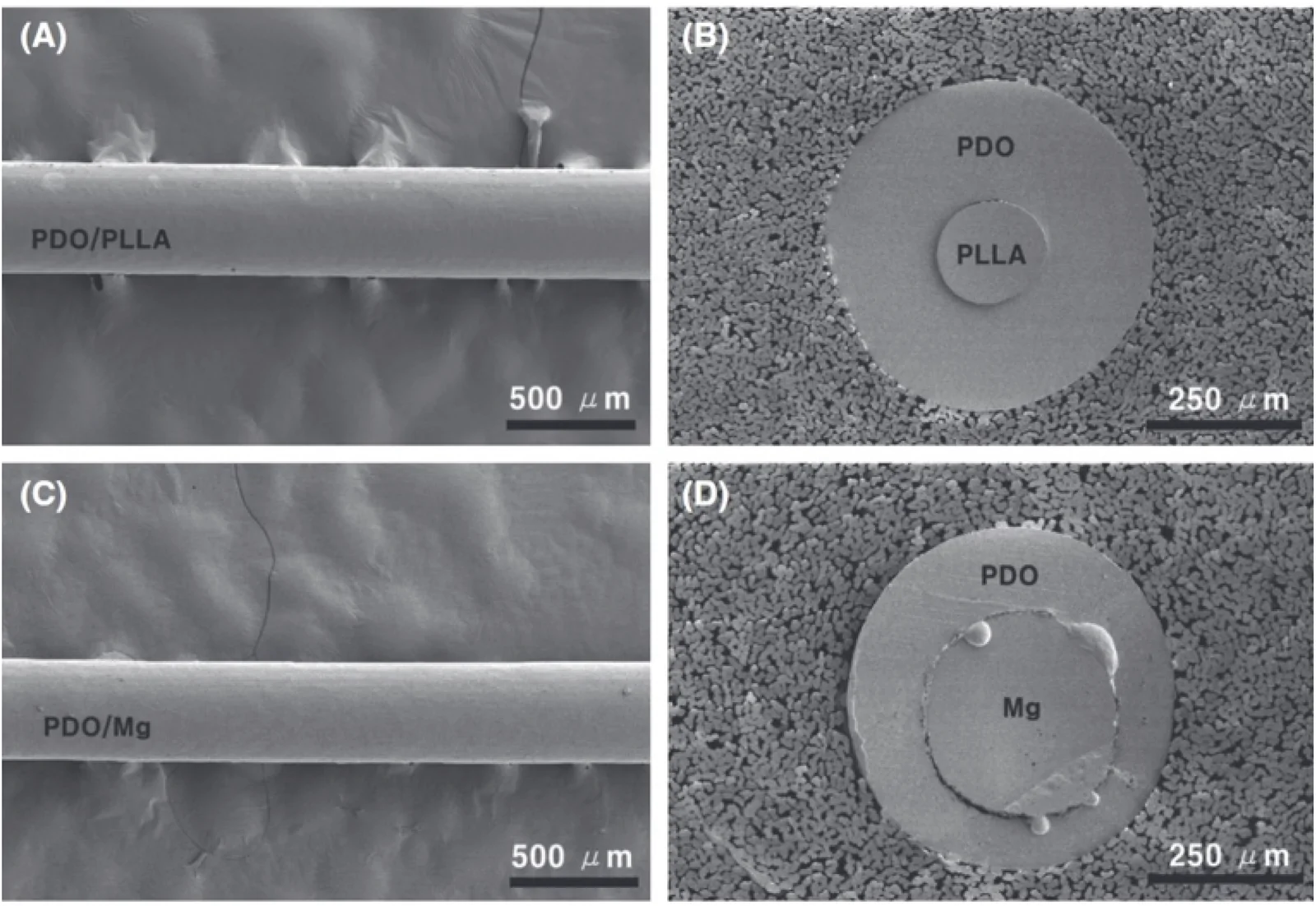
Scanning electron microscopy (SEM) images of PDO/PLLA and PDO/Mg sheath-core monofilaments studied by Kwon *et al.*^5^ (A, C) Surface morphology, with 500 µm scale bars; (B, D) cross-sectional morphology, with 250 µm scale bars. PDO is the outer sheath, surrounding a PLLA core in (B) and a magnesium core in (D). The images show architecture rather than radial-force measurements. PDO/Mg is included as a polymer–metal hybrid comparator. PDO, polydioxanone; PLLA, poly(l-lactic acid); Mg, magnesium.

Flexibility is another important mechanical property that determines the ability of a stent to navigate through the tortuous biliary tract and conform to the natural curvature of bile ducts. Flexibility can be assessed using bending tests with defined span, displacement, and loading conditions. Bioresorbable stents with higher flexibility are easier to deliver and less likely to cause trauma to the bile duct during placement. The flexibility of bioresorbable stents is particularly important for navigating through the distal biliary tract, which has a more tortuous anatomy and is more susceptible to injury during stent placement. Stents with insufficient flexibility may cause perforation or dissection of the bile duct, particularly in patients with strict or angulated anatomy.^2^

Fatigue resistance is also an important consideration, as biliary stents are subjected to repeated mechanical stress from peristaltic movements and changes in intra-abdominal pressure. Fatigue resistance is typically evaluated using a cyclic fatigue tester, which applies repeated bending or compressive loads to the stent until failure occurs. Stents with poor fatigue resistance may fracture or fail prematurely, leading to stent migration or obstruction. Comparisons require the load amplitude, loading mode, test medium, and degradation age to be reported; an initial static strength value cannot substitute for fatigue testing.

The mechanical properties of bioresorbable stents may be optimized through multiple design strategies, including adjusting the polymer composition, modifying the stent geometry, and using composite materials. For example, stents with smaller strut diameters are more flexible but have lower radial force, while stents with larger strut diameters have higher radial force but are less flexible. Stent design parameters such as strut diameter, pitch, and pattern can be optimized to achieve the desired balance between radial force and flexibility. Another approach to tailoring mechanical properties is the use of composite materials, which combine different polymers or incorporate reinforcing fibers to enhance specific properties. In a study by Kwon *et al.*, PDO/PLLA sheath-core stents showed longer radial-force and structural retention than the tested single-material comparators in a bile-flow phantom, demonstrating the benefits of composite designs. The sheath-core design features a PDO sheath that imparts flexibility and a PLLA core that contributes mechanical strength and regulated degradation behavior, resulting in a stent with balanced mechanical performance and controlled degradation kinetics.^5^

Multi-layer designs are another approach to optimizing mechanical properties. These designs typically consist of an inner layer that provides mechanical support and an outer layer that provides drug delivery or biocompatibility enhancement. The inner layer can be designed to have higher radial force and mechanical strength, while the outer layer can be designed to be more flexible and biocompatible. In addition to these design approaches, the mechanical properties of bioresorbable stents can also be tailored through the use of different polymer blends and copolymers. For example, PLCL copolymers, which are composed of lactic acid and caprolactone, can offer greater flexibility than PLA homopolymers, depending on composition and architecture. This makes them suitable for use in self-expandable stent designs that need to conform to the irregular geometry of bile ducts.^2,13^

The mechanical design of bioresorbable biliary stents also needs to consider the effects of degradation on mechanical properties. As bioresorbable polymers degrade, their mechanical properties change, with a gradual loss of strength and stiffness. This requires careful design to ensure that the stent maintains sufficient mechanical integrity to maintain patency until tissue healing is complete. A further key aspect of bioresorbable biliary stent engineering concerns the interaction between the implanted device and the surrounding biological environment. The stent must preserve biliary lumen patency while avoiding excessive irritation or mechanical trauma to the bile duct wall. Achieving this objective requires an appropriate balance between radial force and overall biocompatibility, ensuring that the stent generates sufficient outward pressure to maintain duct openness without exerting forces that may injure adjacent tissues. Structural characteristics of the stent, such as surface morphology and applied coatings, also play an important role in determining tissue responses. For example, smoother surface architectures and coatings with high biocompatibility may mitigate local irritation and reduce inflammatory reactions.

In general, the mechanical design of bioresorbable biliary stents is an engineering compromise among radial force retention, flexibility, fatigue resistance, and degradation. Increasing strut thickness or crystallinity may improve early radial force but can reduce flexibility and delay resorption. Softer or more elastic designs may improve deliverability and duct conformation, yet they may be vulnerable to compression if degradation proceeds faster than tissue recovery. Future designs should therefore report mechanical retention over time in bile-relevant conditions, rather than only initial radial force or static tensile properties.

#### 2.2.2 Degradation kinetics and mechanisms

The degradation kinetics of bioresorbable stents are critical for clinical success, but the term controlled degradation should be interpreted across three distinct levels. *In vitro* degradation in buffered media or bile simulants provides a controlled comparison of mass loss, molecular-weight decline, and mechanical retention. Preclinical *in vivo* degradation adds bile flow, local tissue response, enzymes, bacterial colonization, and device deformation. The real human biliary environment is more variable still, because obstruction severity, infection, bile composition, tumor compression, and prior intervention may all alter the degradation timeline.

The optimal degradation timeline can differ according to the clinical indication being treated. The degradation of bioresorbable polymers occurs primarily through hydrolysis, in which water molecules cleave the ester linkages within the polymer backbone, leading to a decrease in molecular weight and eventual breakdown into smaller molecules. The rate of hydrolysis is determined by multiple parameters, including the chemical structure of the polymer, crystallinity, molecular weight, and the local environment. Polymers with more hydrophilic backbones, such as PGA, generally degrade more rapidly than more hydrophobic polymers such as PLA. This is because hydrophilic polymers absorb more water, which accelerates the hydrolysis process. The crystallinity of the polymer also affects degradation rate, with amorphous regions degrading more rapidly than crystalline regions.^10^

In an *in vitro* bile-flow phantom comparison of several bioresorbable stent prototypes, Kwon *et al.* reported that sheath-core stents composed of PDO/PLLA were able to preserve substantial radial force and structural stability for approximately 16 weeks, whereas PGA and PDO/Mg stents experienced a rapid decline in radial strength and underwent complete degradation within about 6 weeks. In the separate 12 weeks porcine comparison of PDO and PDO/PLLA stents, the PDO/PLLA configuration was associated with a lower frequency of adverse events, including stent fragmentation and biliary stone formation, when compared with devices fabricated from a single material.^5^

The chemical environment of bile also plays a critical role in determining the degradation characteristics of biodegradable stents. Enzyme-mediated acceleration cannot be assumed for every polyester. Xu *et al.* screened PLGA compositions in human bile *in vitro* (Fig. 4). The selected lactide : glycolide ratio of 71 : 29 was then used in rat common-bile-duct repair, where the device disappeared after roughly 4–5 weeks. The rat observation is distinct from the laboratory mass-loss measurements.^12^

**Fig. 4:**
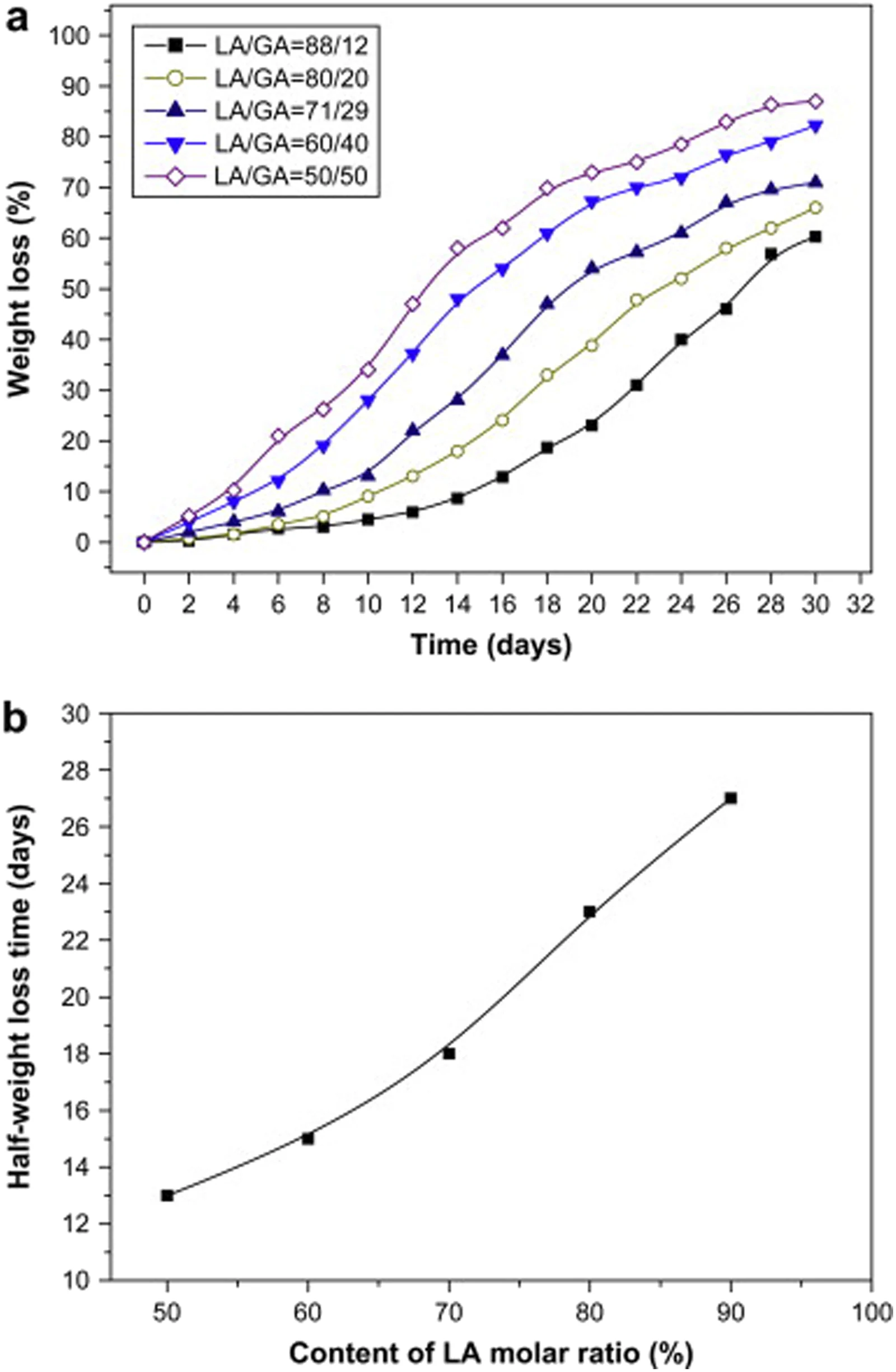
Composition-dependent *in vitro* degradation of PLGA in bile at 37 °C, attributed to Xu *et al.*^12^ (a) Weight loss as a function of incubation time for the indicated lactide : glycolide compositions; (b) half-weight-loss time as a function of lactide content. These are mass-loss endpoints from *in vitro* screening, distinct from the four-to-five-week device-disappearance observation for the selected 71 : 29 formulation in rat bile-duct repair. PLGA, poly(lactic-*co*-glycolic acid); LA, lactide; GA, glycolide.

These examples illustrate why direct translation from *in vitro* half-weight-loss data to clinical patency should be avoided. Polymer mass loss, radial force retention, fragmentation, and tissue healing do not necessarily progress at the same rate. For biliary stents, the most relevant endpoint is not degradation alone, but the interval during which the device maintains sufficient radial support and luminal drainage without causing obstruction, inflammatory injury, or difficult-to-clear fragments.

The degradation process of bioresorbable stents can exhibit two limiting behaviors: bulk degradation where the polymer chains break down throughout the material, and surface erosion where the outer layer of the stent degrades first (Fig. 5). The relative contribution of these behaviors depends on the polymer composition and environmental conditions. Bulk degradation is characteristic of polymers such as PLA and PGA, where water penetrates the entire material and hydrolysis occurs throughout the polymer matrix. This can lead to a rapid loss of mechanical strength once the molecular weight decreases below a critical threshold, even though the mass loss may be minimal. Surface erosion is characteristic of polymers such as poly(ortho esters) and poly(anhydrides), where degradation occurs primarily at the surface of the material. Surface-localized erosion can temporarily preserve a less affected core, but prolonged stent support must be demonstrated rather than inferred from this idealized mechanism.^10^

**Fig. 5:**
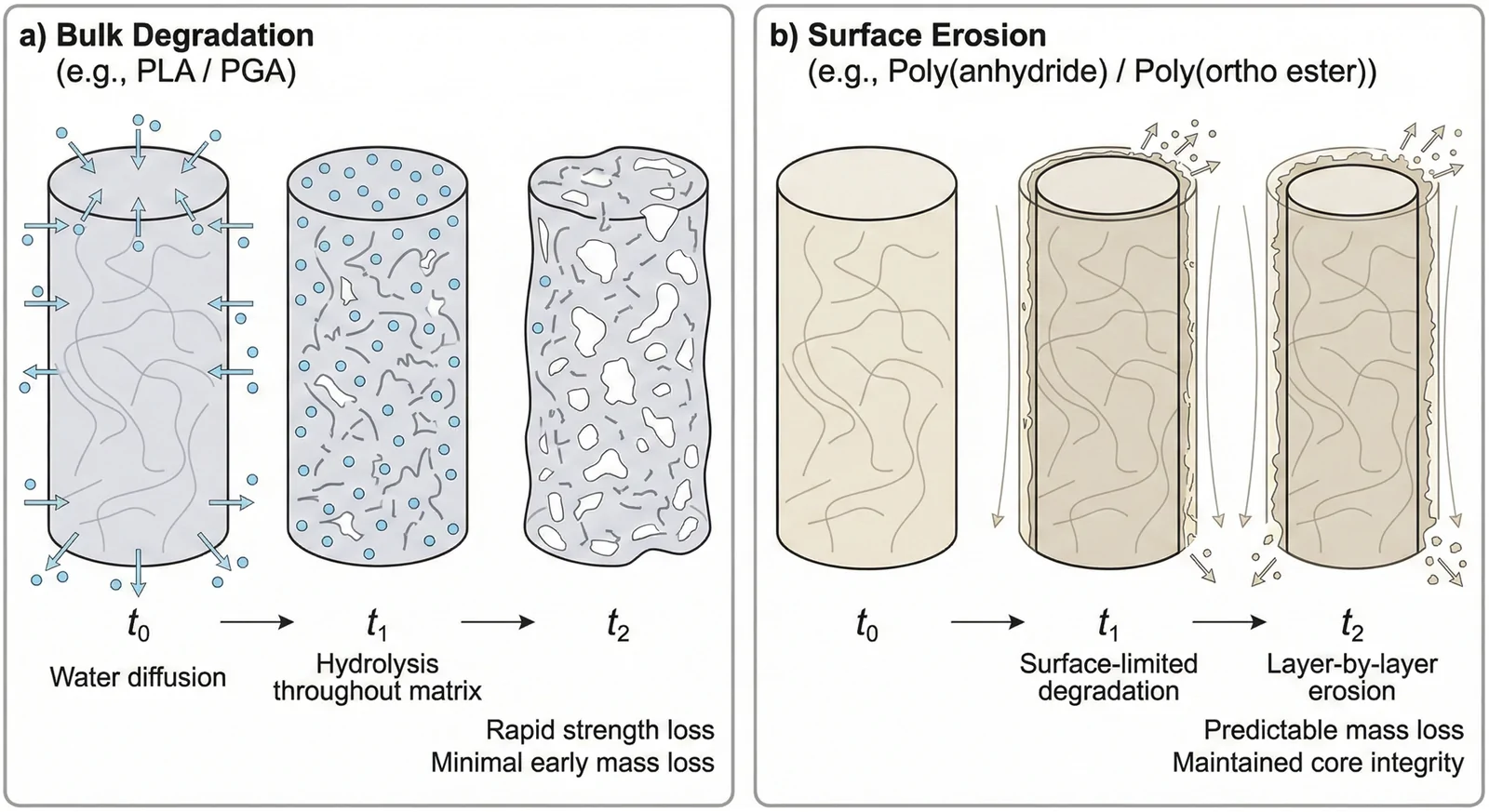
Schematic illustration of the two primary degradation mechanisms of bioresorbable stents: bulk degradation and surface erosion. Water penetration and chain cleavage can reduce strength before appreciable mass loss. The time symbols show conceptual progression, not measured intervals; these are alternative limiting behaviors rather than obligatory consecutive stages.^10^

Bile composition and pH act through different pathways. Water availability, buffering, bile salts, proteins, and lipid deposits can alter wetting, transport, and the local interface. Polymer-derived acidity may differ from bulk bile pH, especially in a stagnant or obstructed segment. Flow can remove soluble products but also adds mechanical exposure. Thus, a bulk-medium pH value alone cannot predict intrapolymer hydrolysis, and bile salts should not be assumed to accelerate every polyester by the same mechanism.^10,12^

Enzymatic activity also requires separation of mechanisms. Polyester hydrolysis can proceed without enzymatic cleavage; an enzyme-mediated contribution must be demonstrated for the polymer and medium used. Bacterial β-glucuronidase is relevant to bilirubin precipitation and sludge formation, which may impair drainage without directly establishing faster scaffold hydrolysis. Biofilm and deposits can change local transport and loading, so microbial colonization should be measured alongside both mass and mechanical changes.^15^

Inflammation and patient-specific factors can further decouple degradation from clinical function. Local tissue injury may change the chemical interface and amplify the response to degradation products. Infection, cholestasis, immunosuppression, prior sphincter intervention, and altered anastomotic anatomy are plausible modifiers of exposure or healing, but the studies discussed do not establish validated patient-level coefficients for their effects on biliary-stent degradation. Their influence should be tested in stratified designs rather than inserted into a universal prediction equation.^3,10^

A bile-relevant test panel should consequently report medium composition and renewal, measured pH, temperature, microbial status, device geometry, and loading. Molecular weight, dry mass, radial-force retention, fragment size, flow resistance, and coating release should be followed concurrently. Matched sterile and colonized conditions, static and flow conditions, and physiologically justified pH ranges would help identify why devices with similar mass loss differ in functional support. These are proposed evaluation priorities, not a validated standard protocol.

#### 2.2.3 Emerging biodegradable metallic and hybrid platforms

Metallic platforms merit separate assessment because their degradation is driven by corrosion rather than polyester hydrolysis. Magnesium offers structural reinforcement and inherent X-ray attenuation, but localized corrosion, hydrogen evolution, and changes in local pH can complicate retention and tissue compatibility. In the PDO/Mg hybrid tested by Kwon *et al.*, rapid *in vitro* deterioration shows why adding a metallic core is not equivalent to achieving prolonged support.^5^

A separate rabbit study by Liu *et al.* evaluated AZ31 magnesium-alloy stents for six months. Remaining device volume was 93.82 ± 1.36% at one month and 30.89 ± 2.46% at three months, with nonuniform corrosion.^16^ This demonstrates that magnesium behavior depends on alloy and architecture: the AZ31 result and the rapidly deteriorating PDO/Mg phantom prototype are not contradictory measurements of the same device. Remaining volume is not a measurement of retained radial force or patency.

Li *et al.* evaluated a Zn–0.8Cu–0.03B alloy biliary stent in rabbits. The structure remained intact for approximately three weeks, with complete degradation reported within eight weeks. *In vitro* antibacterial activity against *Staphylococcus aureus* and *Escherichia coli* was also reported.^9^ These results support preclinical feasibility but do not establish extended support for malignant obstruction or clinical infection control. Zinc corrosion, force retention, and local product burden must be evaluated together.

For a metal or polymer–metal hybrid, testing should couple corrosion-product composition and release to radial-force retention, pitting or fracture, tissue injury, and biliary obstruction. Coatings may delay corrosion initially, but a localized defect can produce nonuniform attack or loss of adhesion. Infection and changing bile chemistry may modify this interface. There is insufficient matched biliary evidence to rank iron-based systems alongside established polymer prototypes; vascular results cannot be transferred without direct biliary evaluation.^9^

### 2.3 Radiopacity and trackability

#### 2.3.1 Importance of radiopacity in biliary stents

Radiopacity is a critical feature for bioresorbable biliary stents to allow visualization during placement and follow-up procedures. The ability to accurately visualize the stent during placement is essential to ensure correct positioning and avoid complications such as stent migration or perforation. Radiopacity also allows for follow-up imaging to evaluate stent position and structural change; imaging disappearance alone does not establish complete resorption.^13,17^

Most biodegradable polymers are inherently radiolucent, meaning they do not absorb X-rays and cannot be visualized using standard radiological techniques. This requires the addition of radiopaque agents to the stent material or coating to enhance visibility. The ideal radiopaque agent should provide high X-ray absorption, biological compatibility, limited leaching, uniform distribution, and minimal disruption of mechanical properties or degradation kinetics. Inorganic fillers such as barium sulfate and tantalum can improve visibility but may aggregate or alter degradation behavior; iodinated copolymers and intrinsically radiopaque polymers can provide more homogeneous visibility but require more complex synthesis and safety validation.^2,17^

#### 2.3.2 Radiopacity enhancement strategies

Several different strategies have been developed to enhance the radiopacity of bioresorbable biliary stents, including the addition of inorganic particles, organic iodine compounds, and radiopaque polymers.^2^

##### 2.3.2.1 Inorganic radiopaque particles

Barium sulfate (BaSO_4_) is one of the most commonly used radiopaque agents for bioresorbable stents. Barium sulfate has high X-ray absorption due to its high atomic number (*Z* = 56 for barium), and it is relatively inexpensive and widely available.^13^

Tanimoto *et al.* developed radiopaque biodegradable biliary stents fabricated from a copolymer of l-lactide and ε-caprolactone (70 : 30) and subsequently coated with barium sulfate. The stents were successfully implanted in the common bile duct of 11 pigs and remained detectable on computed tomography (CT) imaging for up to 2 months after implantation. By 6 months, the stents were no longer visible by CT, an imaging endpoint that does not independently prove complete biological clearance.^13^

However, barium sulfate has several limitations. It is a particulate material that can aggregate within the polymer matrix, leading to non-uniform radiopacity and potential effects on mechanical properties.^2^

Other inorganic radiopaque particles that have been investigated include tungsten, tantalum, and gold. These materials have even higher X-ray absorption than barium sulfate due to their higher atomic numbers, but they are more expensive and require material-specific tissue-response and degradation-product assessment.^2^

##### 2.3.2.2 Organic iodine compounds

Organic iodine compounds are another class of radiopaque agents that have been used in bioresorbable stents. These compounds contain iodine atoms (*Z* = 53) which have high X-ray absorption, and they can be chemically incorporated into the polymer chain or physically mixed with the polymer.^17^

Girard *et al.* developed a radiopaque and resorbable internal biliary stent (RIBS) using a blend of PLA_50_–poly(ethylene glycol) (PEG)–PLA_50_ triblock copolymer and X-ray-visible triiodobenzoate–poly(ε-caprolactone) copolymer (PCL–TIB) (Fig. 6).^17^

**Fig. 6:**
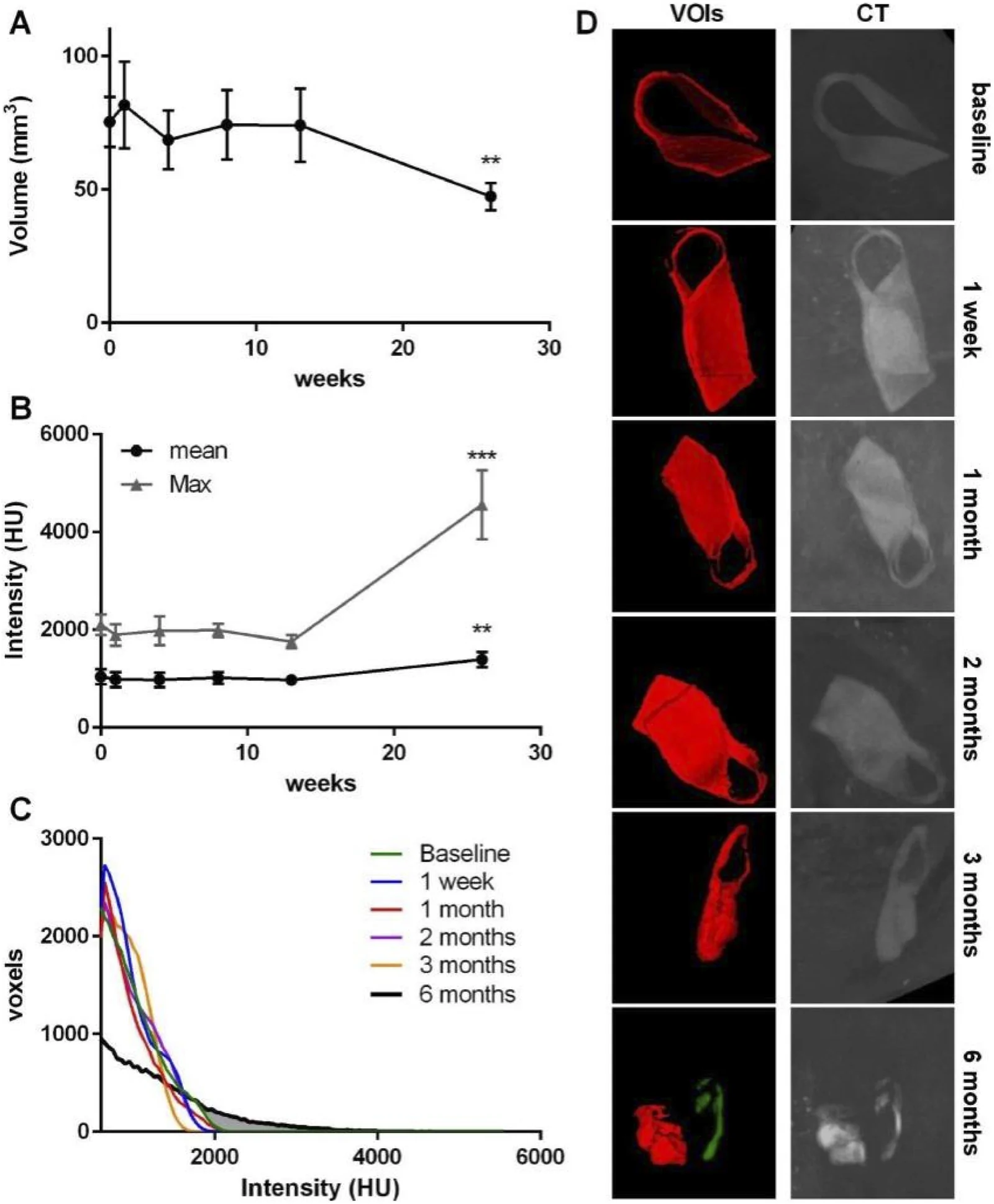
Computed tomography (CT) imaging analysis of rat intraperitoneal implants in Girard *et al.* (A) The volume of the implant from baseline to 6 months. (B) The mean and the maximum signal intensities from baseline to 6 months. (C) The average signal distribution within the volumes of interest (VOIs) at a given time point. All quantitative measurements were calculated using the VOIs. (D) CT scan appearance of the implants over time from the same animal. HU, Hounsfield units. These are not implants in functioning rat bile ducts; six-month imaging visibility does not establish six-month biliary patency.^17^

The PCL–TIB copolymer contains triiodobenzoate groups which provide radiopacity, and it can be blended with other biodegradable polymers to enhance their visibility. Rat intraperitoneal implantation experiments, rather than rat bile-duct stenting, demonstrated that the RIBS remained distinctly detectable under X-ray imaging for a period of up to 6 months, whereas its stiffness decreased by approximately 70% after 4 weeks in the mechanical tests. Histological analyses verified good biocompatibility and progressive resorption, accompanied by only mild inflammatory reactions. Placement feasibility was also examined in a human cadaver; these findings do not establish six-month clinical biliary patency.^17^

One of the main advantages of organic iodine compounds is that they can be incorporated into the polymer matrix with effects on mechanical properties and degradation kinetics that must be measured. However, they may have lower X-ray absorption compared to inorganic particles, and there is potential for leaching of iodine into the surrounding tissue, requiring assessment of local exposure and tissue tolerance.^17^

##### 2.3.2.3 Radiopaque polymers

Another approach to enhancing radiopacity is to use polymers that contain iodine or other heavy atoms within their chemical structure. These materials can serve as the main scaffold or as a coating on a biodegradable polymer frame, reducing the need for separate particulate fillers. At present, a candidate strategy is a hybrid approach in which radiopaque copolymers or well-dispersed low-load fillers provide sufficient procedural visibility while preserving degradation predictability and mechanical stability.^2,17^

The main advantage of radiopaque polymers is that they do not require the addition of separate radiopaque agents, which can simplify the manufacturing process and reduce the potential for aggregation or leaching. However, the synthesis of these polymers can be more complex and expensive, and their mechanical properties and degradation kinetics may be different from traditional biodegradable polymers.^17^

Kim *et al.* evaluated three-dimensional (3D)-printed poly(ε-caprolactone) (PCL)/barium sulfate stents in pigs. Among 11 evaluable stented animals, three devices disappeared on imaging and were interpreted as migrated; three fractures were detected at two months. This provides a direct example of why absent signal and complete resorption must not be equated.^18^

#### 2.3.3 Challenges and controversies

There are several challenges and controversies associated with enhancing the radiopacity of bioresorbable biliary stents. One of the main challenges is finding a radiopaque agent that provides sufficient visibility without significantly affecting the mechanical properties or degradation kinetics of the stent. Another challenge is ensuring that the radiopaque agent is uniformly distributed throughout the stent material, which is essential for consistent visualization. Aggregation of radiopaque particles can lead to non-uniform radiopacity, which can make it difficult to accurately evaluate stent position and patency. The central trade-off is therefore not visibility alone, but visibility without compromising stent function. Increasing radiopaque content can improve fluoroscopic or CT detection, yet excessive filler loading may accelerate water uptake, reduce ductility, weaken fatigue resistance, or leave nondegradable residues. Conversely, fully polymeric radiopaque systems may offer better material integration but require more demanding synthesis, reproducibility testing, and toxicological evaluation before clinical translation.^2,17^

Table 2 summarizes different radiopacity enhancement strategies for bioresorbable biliary stents and their performance characteristics, with the imaging environment, observed duration, and limitations of interpretation.

**Table 2:** Radiopacity enhancement strategies for bioresorbable biliary stents^a^

Strategy	Device and test setting	Observation	Interpretation and remaining challenge	Source
Barium sulfate coating	Lactide–caprolactone stent; porcine bile duct	CT detection at 2 months; no CT detection at 6 months	Visibility demonstrated; disappearance is not chemical proof of complete resorption	13
Iodinated PCL–TIB blend	PLA–PEG–PLA-based implant; rat peritoneal cavity and human cadaver feasibility	Serial CT visibility to 6 months	Non-biliary implantation evidence; establish biliary force retention, contrast, and clearance together	17
PCL/barium sulfate	3D-printed biliary stent; porcine model	CT follow-up; 3/11 devices no longer visible and interpreted as migrated	Imaging disappearance can represent migration; fractures also occurred	18
Degradable metal component	Magnesium hybrid or emerging zinc system	Composition provides an imaging rationale; device-specific validation required	Corrosion and loss of geometry may alter detectability; metallic content does not prove prolonged support	5 and 9

a CT visibility, implant volume, functional drainage, and biological clearance are distinct endpoints. Formulation-specific imaging data should accompany any claimed period of trackability.

## 3. Coating engineering and functionalization strategies

The coating evidence includes fully degradable experimental constructs, degradable coatings on persistent metal frameworks, and concepts transferred from other biomedical applications. These categories are distinguished below and in Table 3. A benefit from a coating on a conventional stent does not establish the safety or effectiveness of a fully bioresorbable coated device.

**Table 3:** Functional coating strategies for bioresorbable biliary stents^a^

Strategy and example	Framework or setting	Evidence stage	Supported conclusion and principal limitation	Source
Paclitaxel membrane	Biliary drug-eluting stent with Pluronic F-127	Preclinical biliary	Serum drug detectable for 28 days with the selected formulation; systemic exposure is not local efficacy	19
Paclitaxel with biodegradable scaffold	Canine biliary reconstruction	Biliary animal	Nine-week drug release and reduced proliferative tissue responses; human benefit unestablished	20
Gemcitabine plus cisplatin/PLCL	Metal framework and PTFE component	Laboratory; mouse tumor; porcine compatibility	More than 30 days *in vitro* release; four-week pig compatibility; whole device is not bioresorbable	23
PVBC–DGL	Experimental biliary coating	Preclinical biliary	Patency 23.4 ± 1.8 weeks with 70% coating; this is not a release interval	15
Silver nanoparticles	Metallic biliary stent	Biliary animal (rabbit)	Less sludge/hyperplasia, but persistent biofilm and greater fibrosis at the highest loading	25
Triamcinolone	Fully covered metal stent	Biliary animal (pig)	Reduced fibrous wall thickness at four weeks; persistent framework and short observation	27
Dexamethasone	Degradable sheath-core braided stent	Biliary animal (swine)	Anti-fibrotic/anti-stricture effect; human dosing and efficacy unestablished	28
Biomimetic/growth-factor interfaces	Candidate resorbable systems	Conceptual/cross-organ extrapolation	Design rationale; direct biliary stent validation needed	29

a Laboratory drug-release time, animal follow-up, and patency duration are separate endpoints. Coating performance on a persistent framework does not establish effectiveness of a fully bioresorbable device.

### 3.1 Drug-eluting coatings for antiproliferative therapy

#### 3.1.1 Principles of drug-eluting coatings

Drug-eluting coatings are an investigational technological approach in bioresorbable biliary stents, aiming to enable localized administration of therapeutic agents to prevent restenosis and support tissue repair. Direct administration of therapeutic agents at the diseased or injured site aims to increase local drug concentrations while limiting systemic exposure and the risk of treatment-related side effects.^7^

The development of drug-eluting coatings therefore requires systematic optimization of multiple parameters, including the selection of appropriate drugs, drug loading levels, release profiles, coating materials, and deposition techniques. An optimal coating strategy should provide sustained and well-controlled drug release during the tissue repair phase, while maintaining strong adhesion to the stent surface and avoiding significant alterations to the stent's mechanical integrity or degradation characteristics.

The drug classes investigated or proposed in biliary stent coatings include antiproliferative agents, anti-inflammatory drugs, antibiotics, and chemotherapeutic agents. Drug selection is determined by the clinical indication, with antiproliferative agents being considered for benign strictures to prevent restenosis, and chemotherapeutic agents being used for malignant strictures to inhibit tumor growth.^7^

#### 3.1.2 Antiproliferative agents

Paclitaxel is a well-known antiproliferative compound that interferes with cell division by stabilizing microtubules, thereby arresting the cell cycle in the G2/M phase. In biliary stents, its most plausible role is to suppress granulation tissue and fibrotic restenosis rather than to act as a definitive local therapy for cholangiocarcinoma. Jang *et al.* developed a paclitaxel-eluting biliary stent incorporating Pluronic F-127 as a sustained-release membrane, in eight pigs, with serum paclitaxel detectable for 28 days using the selected formulation. Serum detection does not establish local efficacy. Shi *et al.* reported reduced granulation tissue and myofibroblast activity in a canine biliary reconstruction model. However, the antitumor effect of paclitaxel against cholangiocarcinoma has been inconsistent, and limited efficacy may reflect tumor biology, insufficient tissue penetration, heterogeneous drug release, or inadequate local dose. Thus, paclitaxel-eluting biliary stents should be presented as promising but not yet clinically established.^19,20^

Shi *et al.* further evaluated PLLA-based stents coated with paclitaxel in a mongrel dog model and found that these devices effectively inhibited the development of granulation tissue and glandular hyperplasia. In addition, reductions in myofibroblast proliferation and excessive extracellular matrix deposition were observed during the healing process. The coated stents maintained sustained paclitaxel release for roughly 9 weeks, and histological analysis revealed decreased granulation tissue formation in groups treated with higher paclitaxel loading levels. Another controversy is the optimal dose of paclitaxel to use in stent coatings. Higher doses may provide better therapeutic efficacy but may also increase the risk of toxicity and inflammatory responses. Lower doses may be safer but may not provide sufficient therapeutic effect.^20^

The translation from local exposure to clinical benefit is uncertain. In a prospective comparison involving 106 patients with malignant obstruction, Jang *et al.* found no significant difference in stent patency or survival between paclitaxel-coated metal stents (60 patients) and covered metal stents (46 patients).^21^ Although this was not a bioresorbable framework, it cautions against assuming that adding an antiproliferative drug necessarily improves drainage. Tissue penetration, dose, and tumor response are possible explanations requiring testing, not established explanations for the negative result.

Sirolimus (rapamycin) and its analog everolimus are another class of antiproliferative agents that have been used in drug-eluting stents. These agents inhibit the mammalian target of rapamycin (mTOR) pathway, which plays a key role in cell proliferation, migration, and survival. Rapamycin has been examined by medication infusion in a rabbit biliary-stricture model, which is different from release from a bioresorbable stent coating.^22^

There are limited direct biliary data on sirolimus or everolimus coatings. More research is needed to determine their optimal dose, release kinetics, and long-term safety and efficacy; cardiovascular experience cannot establish biliary effectiveness.

#### 3.1.3 Chemotherapeutic agents

Gemcitabine belongs to the class of nucleoside analog chemotherapeutic agents and has strong activity against pancreatic cancer and cholangiocarcinoma. Xiao *et al.* fabricated gemcitabine/cisplatin-eluting biliary stents using mixed electrospinning with PLCL as the polymeric carrier on a metal framework with a polytetrafluoroethylene (PTFE) component (Fig. 7), achieving sustained release for more than 30 days *in vitro* and significant inhibition of extrahepatic cholangiocarcinoma cells *in vitro*. These findings support the feasibility of local combination therapy, but they should be interpreted with caution because prolonged exposure to cytotoxic drugs may injure nonmalignant biliary epithelium, delay mucosal healing, promote local inflammation, or generate concentration gradients that are difficult to control in obstructed bile ducts. Future studies should therefore report not only tumor inhibition but also epithelial toxicity, bile duct healing, cholangitis risk, and systemic leakage. The whole device is not bioresorbable; the accompanying mouse tumor and four-week porcine compatibility studies do not demonstrate human antitumor efficacy.^23^

**Fig. 7:**
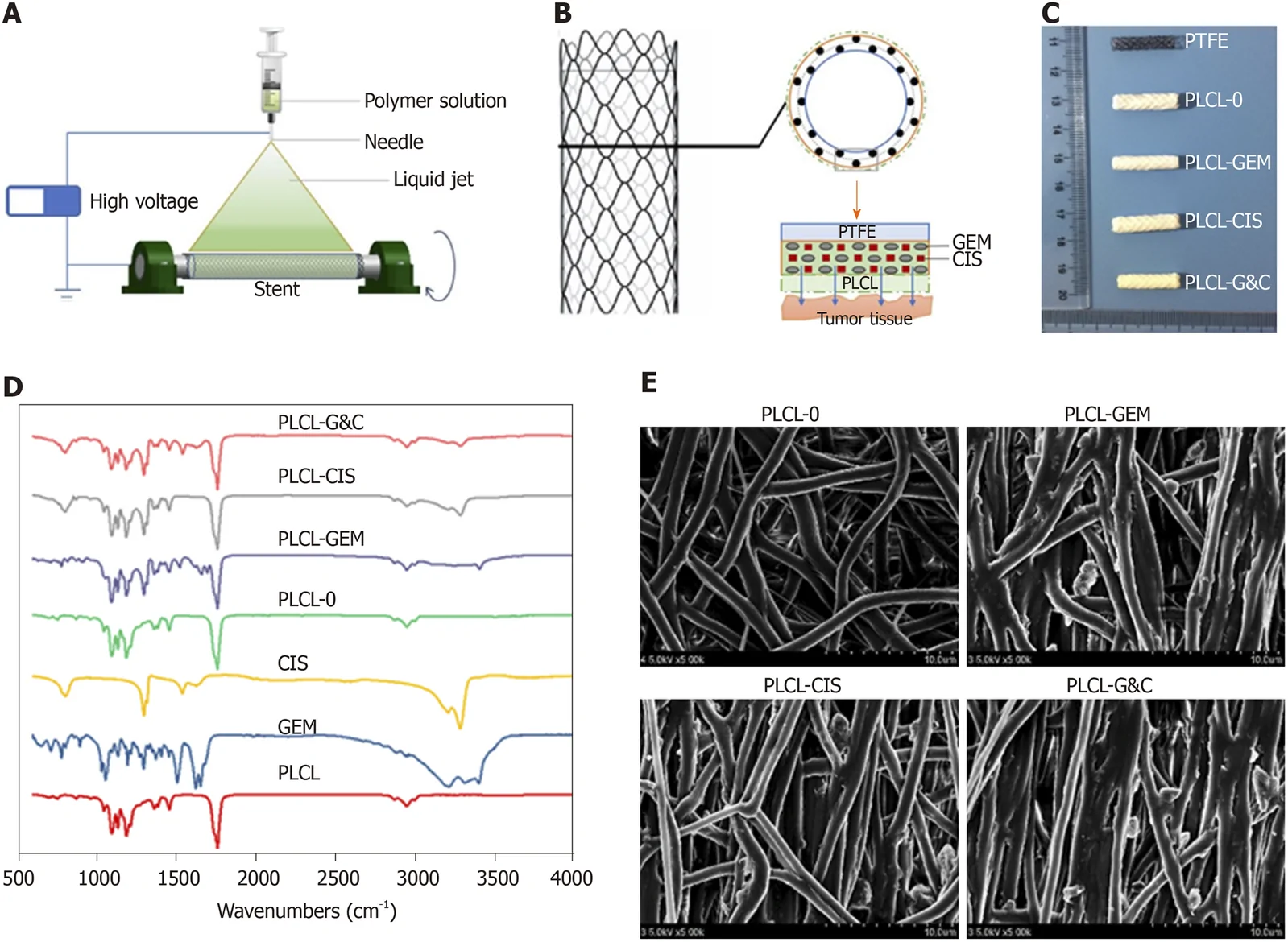
(A) Illustration showing the manufacturing workflow of drug-eluting stents produced using the mixed electrospinning technique; (B) structural design of the optimized gemcitabine/cisplatin-eluting biliary stent; (C) representative photographs of various drug-eluting stent configurations; (D) Fourier transform infrared (FTIR) spectra obtained from prototype drugs and corresponding drug-loaded nanofilms; (E) scanning electron microscopy (SEM) images displaying the microstructures of different drug-eluting nanofilms. The system includes a persistent metal framework and PTFE component with a PLCL drug carrier; the whole device is not bioresorbable. GEM, gemcitabine; CIS, cisplatin; G&C, combined drugs; PLCL-0, unloaded PLCL membrane.^23^

Cisplatin is a platinum-containing anticancer compound that exerts cytotoxic effects by forming cross-links within DNA strands, which disrupts both DNA replication and transcriptional processes. This drug has long been used in the treatment of multiple malignancies, including cholangiocarcinoma, and has therefore been investigated as a potential component of biliary stent-based drug delivery systems. Consequently, the gemcitabine–cisplatin combination has also been incorporated into experimental designs for drug-eluting biliary stents. Despite its therapeutic potential, the clinical use of cisplatin is associated with several limitations, including notable systemic toxicity, the risk of nephrotoxicity and neurotoxicity, and the possibility of acquired drug resistance. These challenges highlight the importance of carefully optimizing cisplatin-based stent delivery systems, and future research should focus on approaches that mitigate toxicity while preserving or enhancing antitumor efficacy.^23^

#### 3.1.4 Coating techniques for drug delivery

Several coating approaches have been explored for constructing drug-eluting biliary stents, including dip coating, electrospinning, spray deposition, and layer-by-layer assembly. Each of these methods exhibits distinct advantages and drawbacks, particularly regarding drug loading capacity, release behavior, coating uniformity, and compatibility with various drug molecules and stent substrates. From a manufacturing perspective, these techniques differ substantially in reproducibility. Dip coating is simple and inexpensive but may produce thickness gradients on braided or complex stent geometries. Electrospinning can create high-surface-area drug reservoirs and combination-drug fibers, but scale-up requires strict control of solvent evaporation, fiber diameter, and batch-to-batch uniformity. Spray coating is often more compatible with clinical-scale production because rotation speed, spray rate, nozzle distance, and layer number can be standardized; however, it still requires in-process monitoring to prevent drug loss and coating defects.^7,23^

Among these techniques, dip coating is considered one of the simplest and most widely implemented strategies for producing drug-eluting stents. During this process, the stent framework is submerged in a polymer solution containing the therapeutic agent and subsequently withdrawn at a controlled velocity, allowing a thin coating layer to form on the device surface. This method offers several practical benefits, such as procedural simplicity, relatively low manufacturing cost, and broad compatibility with diverse drug and polymer systems. Furthermore, coating thickness can be adjusted by altering parameters such as polymer concentration in the solution and the withdrawal speed during coating formation. Despite these advantages, certain limitations remain, including the potential formation of non-uniform coating layers, especially when applied to stents with complex geometries, as well as difficulties in achieving high levels of drug loading. Drug release from dip-coated stents often follows a characteristic profile consisting of an initial burst release phase followed by a slower sustained-release stage, which may not be optimal for all therapeutic scenarios.^7^

Electrospinning is a more advanced coating technique that uses an electric field to produce fine fibers from a polymer solution or melt. The fibers can be deposited directly onto the stent surface to form a porous coating that can incorporate drugs into the fiber matrix. Electrospinning has several advantages over dip coating, including the ability to produce coatings with high surface area and porosity, which can enhance drug loading and control release kinetics. It also allows for the incorporation of multiple drugs into the same coating, enabling combination therapy. Xiao *et al.* used electrospinning to develop gemcitabine and cisplatin-eluting biliary stents with PLCL as the drug carrier. The electrospun coatings showed sustained drug release for more than 30 days *in vitro* and demonstrated significant antitumor activity against cholangiocarcinoma cells. However, electrospinning also has some limitations, including the need for specialized equipment, potential for solvent toxicity, and difficulty achieving uniform coatings on complex stent geometries. The process parameters also need to be carefully controlled to ensure consistent fiber diameter and coating thickness.^23^

Spray coating is also widely applied in the fabrication of drug-eluting stents. In this technique, a solution containing the drug and polymer is atomized into microdroplets and deposited onto the rotating stent surface, which is typically rotated to ensure uniform coverage. Spray coating has several advantages, including its ability to form homogeneous coatings even on complex stent structures, its compatibility with a wide range of drugs and polymers, and its ability to control coating thickness by adjusting the spray parameters. It also allows for the production of multi-layer coatings with different drug release profiles. However, spray coating also has some limitations, including possible drug loss during atomization, difficulty achieving high drug loading concentrations, and the risk of coating irregularities if spray parameters are not precisely controlled. The release kinetics from spray-coated stents are also typically characterized by an initial burst release followed by slower sustained release.^7^

Fig. 8 illustrates dip coating, electrospinning, and spray coating as representative drug-eluting stent coating techniques. For translational manufacturing, spray coating and other automated layer-based processes offer a potential path to reproducible coating uniformity, whereas electrospinning is especially attractive when high drug loading, porous architecture, or multi-drug release is required.

**Fig. 8:**
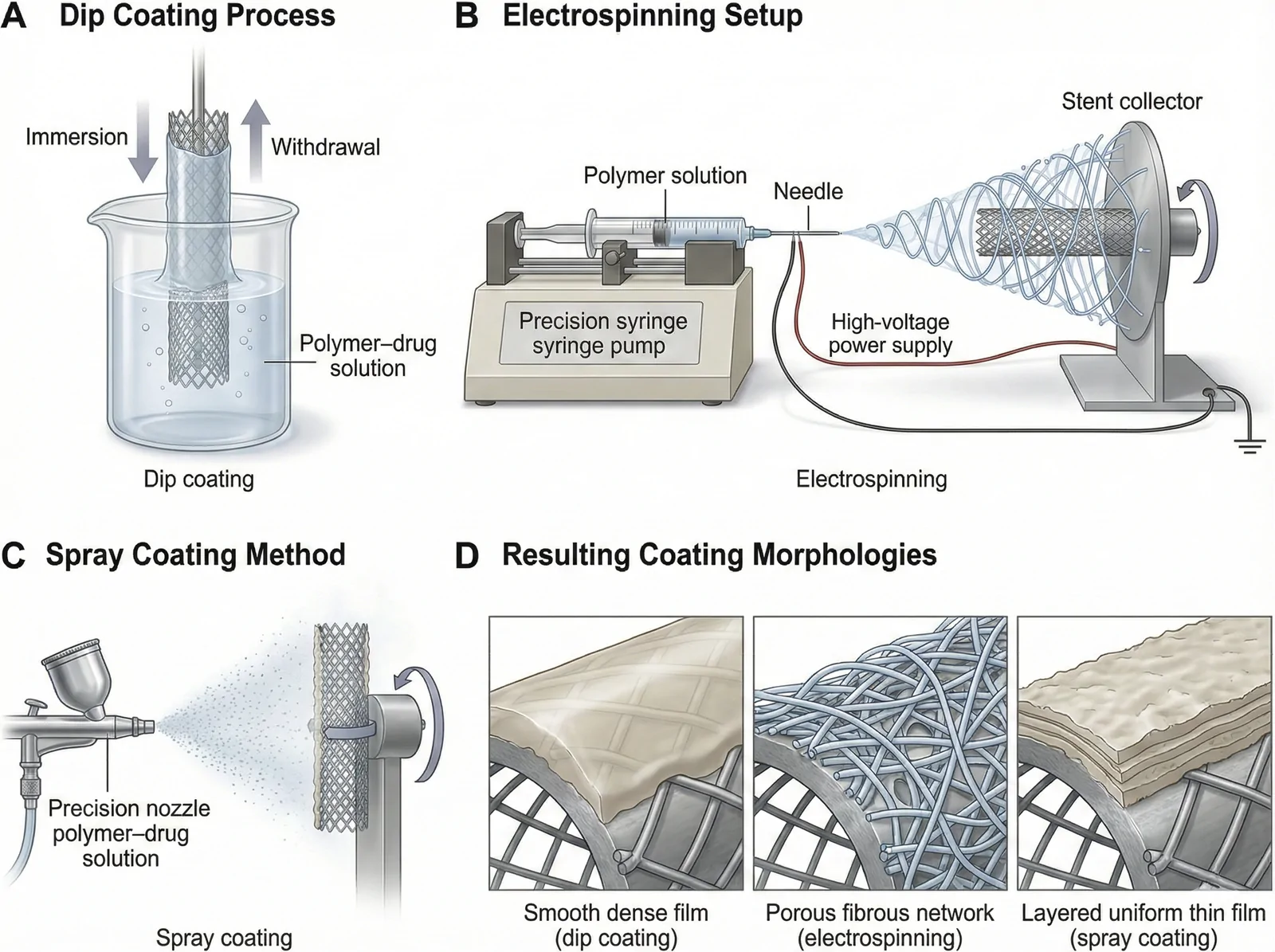
Schematic representation of drug-eluting stent coating techniques. (A) Dip coating process, (B) electrospinning setup, (C) spray coating method, (D) resulting coating morphologies.

### 3.2 Anti-biofouling and anti-inflammatory coatings

#### 3.2.1 Anti-biofouling coatings

Biofouling, defined as the accumulation of proteins, bile components, microorganisms, and debris on the stent surface, is a major cause of stent occlusion and infection. Anti-biofouling coatings can work through several mechanisms, including hydration-layer formation, reduced surface energy, enzymatic inhibition of biliary sludge deposition, contact-killing antibacterial activity, and release of antimicrobial agents. In bile-rich environments, however, durability is a central limitation because coating swelling, bile salt adsorption, bacterial biofilm maturation, and polymer degradation can progressively reduce anti-adhesive or antimicrobial activity.^15,24^

Biliary sludge deposition is a common complication of biliary stents, particularly in patients with cholestasis or impaired bile flow. This process is mediated by β-glucuronidase, an enzyme that hydrolyzes bilirubin glucuronide to form unconjugated bilirubin, contributing to calcium bilirubinate precipitation and biliary sludge. Zhang *et al.* reported a bile duct stent coated with a newly designed polyvinyl benzyl chloride–d-glucaro-1,4-lactonate polymer (PVBC–DGL) aimed at inhibiting biliary sludge accumulation. d-Glucaro-1,4-lactonate functions as a selective competitive inhibitor of β-glucuronidase, thereby blocking the hydrolysis of bilirubin glucuronide and consequently decreasing the formation of biliary sludge. The stents with 70% PVBC–DGL showed significantly prolonged patency time (23.4 ± 1.8 weeks; *n* = 5) compared to uncoated stents (11.2 ± 2.1 weeks; *n* = 5) and 0% coating controls (10.5 ± 2.5 weeks; *n* = 4) in a pig model. The coated stents also showed significantly less biliary sludge deposition and biofilm formation compared to uncoated stents. These are patency durations, not measured drug-release periods, and do not establish biodegradation of the complete framework.^15^

Bacterial colonization of biliary stents can lead to cholangitis, stent occlusion, and systemic infection. Common pathogens include *Escherichia coli*, *Klebsiella pneumoniae*, and *Enterococcus faecalis*. Silver-containing coatings provide broad-spectrum antimicrobial activity by damaging bacterial membranes, suppressing DNA replication, and disrupting metabolic pathways. Their main advantages are immediate bactericidal action and broad activity, whereas limitations include possible ion leaching, local irritation, uncertain long-term durability, and incomplete protection once mature biofilm has formed. Antibiotic and antimicrobial-peptide coatings may offer more targeted activity, but resistance selection, release exhaustion, and stability in bile remain important barriers.^24,25^

Park *et al.* evaluated silver-nanoparticle-coated metallic stents in rabbit extrahepatic bile ducts, with successful placement in 22 of 24 animals. Coated groups had less sludge and tissue hyperplasia, but biofilm remained present in all groups, and the highest silver loading increased submucosal fibrosis relative to the other coated groups.^25^ This directly illustrates the balance between surface activity and local tolerance. The persistent metal substrate also limits transfer to a fully bioresorbable device. Qiao *et al.* combined Si/Sr/Ag-substituted hydroxyapatite with titania nanotube arrays on titanium and reported antibacterial activity against *Staphylococcus aureus* alongside compatibility with osteoblastic cells.^26^ This orthopaedic coating study supports evaluating antimicrobial activity together with host-cell responses; biliary epithelial safety and performance in bile require separate testing.

Antibiotic coatings are a proposed approach to preventing bacterial colonization of biliary stents. These coatings typically contain antibiotics such as gentamicin, ciprofloxacin, or vancomycin, which are released in a controlled manner to provide local antimicrobial activity. However, there is concern about the development of bacterial resistance to antibiotics used in stent coatings, which could limit their long-term efficacy.^7^

Antimicrobial peptides (AMPs) represent an emerging category of antimicrobial molecules with broad-spectrum activity against bacteria, fungi, and viruses. AMPs are short peptide sequences capable of damaging bacterial cell membranes, and their resistance-selection potential depends on the peptide and exposure. These are candidate approaches; direct biliary release, safety, and patency evidence is needed before clinical benefit can be assigned.^7^

#### 3.2.2 Anti-inflammatory coatings

The implantation of a biliary stent triggers an inflammatory response in the surrounding tissue, which can lead to the formation of granulation tissue and restenosis. Anti-inflammatory coatings aim to reduce this inflammatory response by delivering anti-inflammatory agents to the site of implantation.

Corticosteroids are investigated anti-inflammatory agents in stent coatings. These agents inhibit the production of inflammatory cytokines and reduce the recruitment of inflammatory cells to the site of injury. Jang *et al.* tested triamcinolone-eluting fully covered metal stents in 12 mini-pigs with induced benign strictures. At four weeks, the 30 mg group had a lower fibrous wall thickness than controls (468.5 *versus* 773.1 µm; *P* = 0.037). Histological improvement with this metallic platform is not a human patency result.^27^ However, there is controversy regarding the long-term safety of corticosteroid-eluting stents. Prolonged use of corticosteroids can have several side effects, including immunosuppression, osteoporosis, and hyperglycemia. While the localized delivery of corticosteroids through stent coatings may reduce systemic side effects, there is still concern about the potential for local tissue effects such as delayed wound healing.

More directly relevant to complete biodegradability, Lee *et al.* developed dexamethasone-impregnated sheath-core monofilaments braided into biliary stents and reported anti-fibrotic and anti-stricture effects in a swine model. The study provides preclinical evidence for integrating steroid delivery within a degradable framework, but it does not establish a clinically validated dose, support interval, or benefit in immunosuppressed patients.^28^

For a degradable device, anti-inflammatory activity should be assessed alongside epithelial continuity, infection, stricture recurrence, and the response to fragments or corrosion products. A coating that lowers an inflammatory marker while delaying healing may not improve drainage. Appropriate controls include the drug-free coated scaffold as well as the uncoated scaffold, because the coating polymer itself can change surface and degradation behavior.

Non-steroidal anti-inflammatory drugs (NSAIDs) represent another category of anti-inflammatory compounds considered for incorporation into stent coatings. These agents act by inhibiting cyclooxygenase (COX) enzymes, which are involved in the production of prostaglandins and other inflammatory mediators. However, NSAIDs also have several limitations, including their potential for causing gastrointestinal irritation, renal toxicity, and bleeding complications. While the localized delivery of NSAIDs through stent coatings may reduce systemic side effects, there is still concern about the potential for local tissue effects. Direct evidence for a biliary drug-eluting scaffold is required before assigning efficacy to this drug class.

Immunomodulatory agents are candidate anti-inflammatory therapies intended to act on specific elements of the immune system. These agents include monoclonal antibodies, cytokines, and small molecule inhibitors that regulate inflammatory pathways through targeted immune modulation. However, immunomodulatory agents are typically more expensive than traditional anti-inflammatory agents, and they may have a higher potential for causing adverse effects such as immunosuppression. More research is needed to determine the optimal use of these agents in biliary stent coatings. Their discussion here is a research rationale rather than evidence of validated biliary coating performance.

Table 3 summarizes different functional coating strategies for bioresorbable biliary stents and their therapeutic effects, distinguishing framework, evidence stage, measured endpoint, and limitations. Overall, antiproliferative coatings are conceptually aligned with benign restenosis and hyperplasia control, chemotherapeutic coatings with malignant obstruction, antimicrobial and anti-biofouling coatings with infection or sludge-prone settings, and biomimetic coatings with tissue repair. No single coating category is universally superior; the design choice depends on whether the dominant clinical problem is fibrosis, tumor growth, bacterial colonization, epithelial injury, or long-term manufacturability.

### 3.3 Biomimetic and tissue-engineering coatings

Biomimetic coatings aim to mimic the natural extracellular matrix to promote cell adhesion, proliferation, and differentiation. Candidate coatings include extracellular matrix proteins, growth factors, or peptides that can enhance tissue regeneration. The goal of biomimetic coatings is to create a microenvironment that supports the natural healing process, promoting the regeneration of healthy bile duct tissue rather than simply preventing restenosis. Extracellular matrix proteins such as collagen, fibronectin, and laminin are commonly used in biomimetic coatings due to their ability to promote cell adhesion and migration. Specific adhesion sequences, including arginine–glycine–aspartic acid (RGD) in proteins such as fibronectin, interact with cell surface receptors. For example, Xiang *et al.* developed gelatin/heparin-coated polyurethane composite fibers for small-caliber vascular grafts. This vascular-graft study provides cross-organ design inspiration but does not establish biliary-stent performance.^29^

Collagen surface modification is a candidate strategy to improve the biocompatibility of biodegradable biliary stents. Fibronectin is a large glycoprotein that plays an essential role in cellular adhesion, migration, and proliferation. Potential benefits of these interfaces for biliary epithelial repair require direct testing; cell attachment alone does not establish drainage, resistance to biofilm, or stricture resolution. Mussel-inspired polydopamine offers another route to surface functionalization. Fu and Yu describe its formation on diverse organic and inorganic substrates and capacity for secondary modification.^30^ These properties provide a rationale for an anchoring layer in biomimetic coating design, while biliary epithelial responses, stability in bile, and effects on scaffold degradation require direct evaluation.

Laminin is another important extracellular matrix protein that is highly enriched in basement membrane structures. Laminin-mediated adhesion is a design rationale for candidate tissue-interactive interfaces. Growth factor delivery has also been explored as a strategy to accelerate tissue repair. Vascular endothelial growth factor (VEGF) is a potent angiogenic factor that induces the formation of new blood vessels, which is essential for tissue repair and regeneration. These biological functions do not establish a safe biliary-stent dose or release window.

Heparin-conjugated PLGA microspheres are candidate affinity-based depots for growth-factor delivery, leveraging heparin's high binding affinity to VEGF to modulate release kinetics. Such strategies are conceptually attractive for bioresorbable biliary stents aimed at accelerating tissue repair through enhanced angiogenesis and epithelialization, but this rationale does not establish direct large-animal biliary-stent efficacy. Another challenge is determining the optimal dose and release kinetics for growth factors. Too little growth factor may not provide sufficient therapeutic effect, while too much may cause excessive angiogenesis, fibrosis, or even tumor promotion. The cost of growth factors is also a major consideration for clinical translation. Growth factors are typically expensive to produce, which can increase the cost of bioresorbable stents significantly. This may limit their widespread use, particularly in healthcare systems with limited resources.^8^

Peptide coatings are another approach to biomimetic surface modification. These coatings use short peptide sequences that mimic the active sites of extracellular matrix proteins, potentially simplifying surface presentation, although stability and cost depend on the formulation. One example is the use of RGD peptides, which are derived from the cell adhesion sequence found in many extracellular matrix proteins. RGD peptides have been shown to promote cell adhesion and proliferation in a variety of cell types, but transfer to a biliary stent requires direct testing. Another example is the use of heparin-binding peptides, which can bind to growth factors and promote their localization to the stent surface. These peptides can be used to create a “growth factor depot” on the stent surface, aiming to provide sustained release of growth factors over time. Fig. 9 illustrates proposed mechanisms of action for different functional coatings, showing how each type of coating interacts with the surrounding tissue to support intended therapeutic effects.

**Fig. 9:**
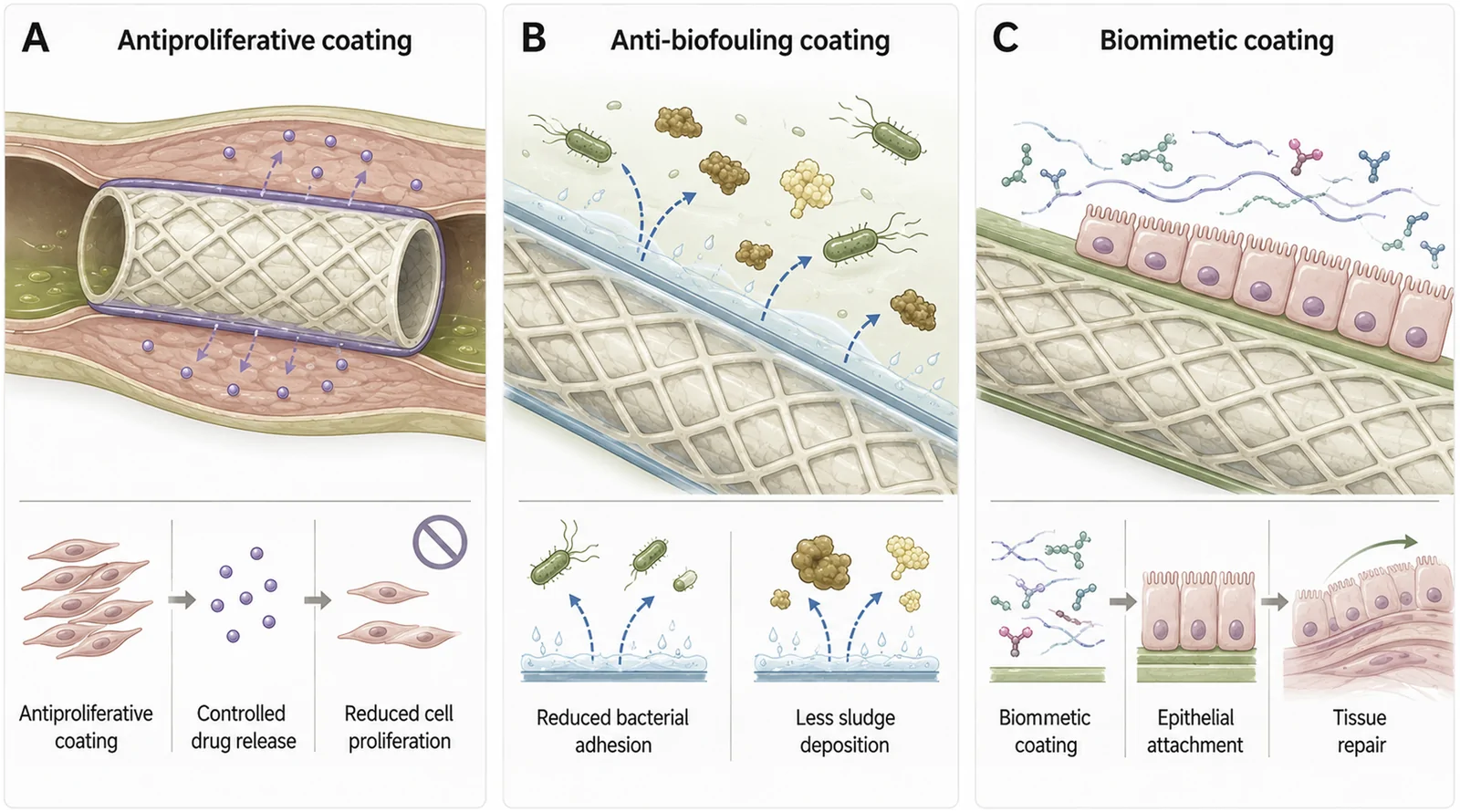
Proposed mechanisms of action for different functional coatings on bioresorbable biliary stents. (A) Antiproliferative coatings aimed at suppressing fibroproliferative responses, (B) anti-biofouling coatings aimed at reducing bacterial adhesion, (C) biomimetic coatings aimed at supporting epithelial cell attachment and proliferation. The chain-like and branched symbols in panel (C) represent generic extracellular-matrix components and cell-adhesion ligands; they are schematic icons, not molecular structures or identified coating constituents. The arrows indicate intended biological effects rather than measured outcomes.

## 4. Clinical translation and outcomes

### 4.1 Preclinical studies and animal models

Preclinical studies play a crucial role in assessing the safety and therapeutic performance of bioresorbable biliary stents prior to clinical application in humans. Various animal models have been used, including pigs, dogs, rabbits, and rats, each offering specific experimental advantages as well as certain limitations. Porcine models are commonly used due to their anatomical similarity to humans, particularly regarding bile duct dimensions and physiological characteristics. Siiki *et al.* compared biodegradable biliary stents (BDBS) with covered self-expandable metal stents (CSEMS) in a pig model of ischemic benign biliary strictures. The results showed that BDBS restored galectin-2 expression toward levels observed in normal bile ducts, while CSEMS therapy resulted in persistently low levels. Transgelin expression remained low after BDBS treatment but was elevated following CSEMS therapy, suggesting different effects on tissue healing.^31^

Grolich *et al.* evaluated self-expandable PDO biliary stents in 23 pigs. The stents successfully achieved full self-expansion in every case, with no bile duct obstruction or post-surgical complications. Macroscopic evaluation revealed progressive changes in the stent structure, including lightening of stent color at 2 weeks, partial disintegration at 8 weeks, and complete absorption at the 13- and 20 weeks assessments. Histologic evaluation showed a mild-to-moderate inflammatory response within the lamina propria, with no clinical consequences.^14^

Canine models have also been used for evaluating bioresorbable biliary stents. Shi *et al.* used mongrel dogs to evaluate paclitaxel-coated PLLA stents for preventing benign biliary stricture after biliary reconstruction. The results showed that the paclitaxel-coated stents reduced granulation tissue proliferation and myofibroblast activity, with no significant differences in liver function among groups. Table 4 summarizes key preclinical studies evaluating bioresorbable biliary stents, including animal models, stent materials, and main outcomes.^20^

**Table 4:** Summary of key preclinical studies evaluating bioresorbable biliary stents

Study	Setting	Platform	Observation and limitation
Kwon *et al.*^5^	Bile-flow phantom; then 12 pigs with 24 PDO or PDO/PLLA stents	PDO, PGA, PDO/PLLA, PDO/Mg screened *in vitro*	16 weeks retention and 6 weeks complete degradation concern the phantom; animal morphology persisted about 12 weeks
Zhang *et al.*^11^	Porcine bile duct	PLA	Ten of 15 assigned animals analyzed; reported patency differs from the filament-breakage degradation criterion
Grolich *et al.*^14^	23 pigs	Self-expanding PDO	Complete absorption observed at 13- and 20 weeks assessments; animal safety and tissue response only
Siiki *et al.*^31^	Porcine ischemic strictures; tissue comparison at 6 months	Biodegradable *versus* covered metal stents	Differences in healing markers; small tissue groups and no human comparative endpoint
Shi *et al.*^20^	Canine biliary reconstruction	Paclitaxel-associated biodegradable stent	Reduced proliferative tissue responses; no demonstration of human stricture resolution
Girard *et al.*^17^	Rat peritoneal implantation; human cadaver feasibility	Radiopaque PLA–PEG–PLA/PCL–TIB	Visibility to 6 months; not a living bile-duct implantation study
Xiao *et al.*^23^	Cell testing, mouse tumor model, and pig bile duct	Drug-loaded PLCL with metal/PTFE components	Drug delivery and compatibility; whole device is not bioresorbable

### 4.2 Human clinical trials and case series

The clinical translation of bioresorbable biliary stents has been relatively slow compared with cardiovascular applications, and the available human evidence includes small cohorts, early feasibility reports, and larger observational registries. Current studies are valuable for demonstrating technical feasibility, but they are insufficient to establish durable superiority over plastic stents or SEMS across all indications.

Sánchez-Cabús *et al.* conducted an early clinical investigation evaluating resorbable biliary stents (RBS) in 12 living donor liver transplantation (LDLT) recipients. The results showed no adverse events related to stent implantation, retention, or degradation. After one early retransplantation, four of 11 evaluable patients presented with biliary complications: 1 case of biliary leak, 1 case of biliary stenosis, and 2 cases presenting both conditions. Patient and graft 1 year survival was 100% and 91.7%, respectively. Patient and graft survival are distinct from stent patency.^32^

Janousek *et al.* described their initial experience with absorbable biliary stents in 10 liver transplant patients, with 5 randomized to stent placement and 5 to control. The results showed good patency in all 5 patients who received stents, with no signs of biliary leakage or stone formation. The stents were completely absorbed with no adverse effects.^33^

Siiki *et al.* conducted a comparative clinical evaluation examining the use of biodegradable biliary stents *versus* traditional plastic stents in the treatment of cystic duct leakage following cholecystectomy. The study enrolled a total of 32 patients, among whom 8 individuals were treated with biodegradable biliary stents (BDBS) and the remaining 24 received standard plastic stents. Clinical outcomes indicated that BDBS therapy showed similar observed short-term safety proportions to plastic stents, with both groups exhibiting a 13% readmission rate and a 13% adverse-event rate within 30 days in each group. A key procedural difference was that patients receiving plastic stents required a second endoscopic intervention for device removal, whereas those treated with BDBS avoided repeat endoscopy due to spontaneous stent degradation. In addition, patients treated with biodegradable stents exhibited markedly lower drainage output, measuring 83 mL compared with 330 mL in the plastic stent group (*P* = 0.002). This small nonrandomized comparison does not establish equivalence or formal cost-effectiveness.^4^

Vaz *et al.* reported early clinical outcomes from a cohort of 9 patients with benign biliary strictures who underwent treatment using biodegradable stents. Both procedural and therapeutic success rates reached 100% in this preliminary series, although one patient experienced stent migration that did not require additional intervention. The average duration of the procedure was approximately 45 minutes, and 77.7% of patients were managed under local anesthesia combined with sedation. Follow-up over a median period of 11 months revealed no evidence of restenosis, providing preliminary short-term observations. Three additional non-traversable cases required surgery, which limits generalization of the treated-group success rate.^34^

Larger experiences broaden the evidence beyond isolated pilot studies. Anderloni *et al.* enrolled 38 patients in a prospective single-center feasibility study, placing 53 biodegradable stents: 34 biliary and 19 pancreatic. Five premature migrations (9.4% of all stents) were reported. The mixed-organ population and degradation-focused endpoint limit biliary-specific effectiveness conclusions.^35^ Battistel *et al.* studied 18 liver transplant recipients with refractory anastomotic strictures treated percutaneously, reporting complete resolution in 72% at a median 27.2 months.^36^

The prospective multicenter BiELLA registry enrolled 159 patients with benign strictures and reported primary patency estimates of 86.7%, 79.6%, and 78.9% at 12, 36, and 60 months, respectively. Mean follow-up was 45.4 months.^37^ This provides longer-term observational evidence, although patient selection and the absence of a randomized comparator prevent attributing superiority to biodegradability alone.

The optimal indication is likely to differ by disease context. Benign strictures and bile leaks may benefit most from temporary support that avoids a second endoscopy. Post-transplant anastomotic strictures require careful balancing of support duration, infection risk, and anastomotic healing. Malignant obstruction may require longer patency, stronger radial force, and local antitumor delivery, but these proposed advantages must be demonstrated against established SEMS in adequately powered studies.

### 4.3 Comparative clinical evidence and economic considerations

The comparative effectiveness of bioresorbable biliary stents *versus* traditional stents is still being evaluated, with limited data available from randomized controlled trials.

The study by Siiki *et al.* showed that bioresorbable biliary stents avoided planned endoscopy for removal in a small bile-leak comparison; this small comparison does not establish general cost-effectiveness. The study found lower total drain output, but unplanned rescue procedures remain possible.^4^ In chronic-pancreatitis-associated benign strictures, a randomized trial of conventional FCSEMS *versus* multiple plastic stents found similar stricture resolution and fewer endoscopic procedures with FCSEMS. This is an indication-specific comparator for future bioresorbable trials, not evidence of bioresorbable-device superiority.^38^

Avoiding repeat endoscopy may improve patient convenience and reduce procedure-related burden, but quality-of-life benefits should be described cautiously because the cited clinical evidence does not establish benefits using standardized patient-reported outcome measures. However, device price may be a barrier to widespread adoption. Cost-effectiveness will depend on device price, complication reduction, avoidance of repeat endoscopy, hospital resource use, and the clinical indication being treated. Table 5 summarizes the key clinical outcomes from human studies of bioresorbable biliary stents.

**Table 5:** Summary of clinical outcomes from human studies of bioresorbable biliary stents^a^

Study/setting	Population	Reported outcome	Evidence limitation
Sánchez-Cabús *et al.*^32^ Living-donor transplantation	12 recipients	4/11 evaluable recipients had biliary complications after 1 early retransplantation; 1 year patient/graft survival 100%/91.7%	Survival is not stent patency; small selected cohort
Janousek *et al.*^33^ Transplantation pilot	10 total; 5 stented and 5 controls	Favorable patency and absorption observations in treated patients	Treated denominator is 5; inadequate for reliable comparative safety
Siiki *et al.*^4^ Post-cholecystectomy bile leak	32 total; 8 biodegradable and 24 plastic	Readmission and 30 days adverse-event rates each 13% in both groups; planned removal avoided with biodegradable stents	Small nonrandomized comparison; similar observed proportions do not establish equivalence
Vaz *et al.*^34^ Benign strictures	9 treated; 3 additional non-traversable cases required surgery	Technical/clinical success in all 9; 1 migration; median follow-up 11 months	Uncontrolled series; limited precision and long-term generalizability
Anderloni *et al.*^35^ Endoscopic single-center study	38 patients; 53 stents (34 biliary, 19 pancreatic)	5 premature migrations (9.4% of all stents); degradation was the main endpoint	Mixed biliary/pancreatic denominator; not a definitive biliary-effectiveness trial
Battistel *et al.*^36^ Refractory post-transplant strictures	18 adults; percutaneous treatment	72% complete resolution; median follow-up 27.2 months	Retrospective selected cohort; limits transfer to first-line endoscopic care
De Gregorio *et al.*^37^ Multicenter BiELLA registry	159 enrolled; mean follow-up 45.4 months	Primary patency 86.7%, 79.6%, 78.9% at 12, 36, 60 months	Prospective observational data; no randomized comparator

a Study-specific denominators and endpoints are retained. Absence of a reported adverse event in a small cohort does not establish absence of risk. Human evidence here does not establish effectiveness of fully resorbable drug-eluting stents for malignant obstruction.

### 4.4 Indication-specific synthesis

Indications differ in the biological task that support must accomplish. A bile leak requires drainage while the leak closes; a fibrotic stricture requires sustained remodeling; transplant anatomy adds ischemia, altered access, and anastomotic risks. Malignant obstruction introduces continuing external compression and tumor growth. Table 6 brings these requirements together and explicitly separates human evidence from proposed applications. Support targets should follow the clinical healing or drainage task rather than generic polymer resorption ranges.

**Table 6:** Indication-specific design and evidence synthesis^a^

Indication	Functional task and support target	Material/coating implications	Human evidence and unresolved requirement
Postoperative bile leak	Maintain drainage through leak closure; no universal calendar duration	Conformable temporary scaffold; prevent early migration or blockage	Small comparative bile-leak experience; prove durable closure and acceptable rescue burden^4^
Benign fibrotic stricture	Maintain drainage during remodeling; conventional FCSEMS treatment for chronic-pancreatitis strictures may last 6–12 months	Sustained force retention without injury; antiproliferative coating remains exploratory	Clinical polymer series; match the disease-specific comparator and assess recurrence after degradation^34,35,39^
Post-transplant anastomosis or stricture	Distinguish prophylactic support from treatment of established stenosis	Control force, fragments, infection risk, and access for rescue	Human pilot and percutaneous cohort data; these settings cannot be pooled indiscriminately^32,33,36^
Malignant obstruction	Maintain drainage despite progressive compression and tumor growth	Persistent force and optional local therapy; epithelial toxicity and systemic leakage matter	Fully bioresorbable drug-eluting applications remain preclinical or proposed; metal/PTFE drug carriers supply adjacent evidence, not full-device validation^3,23^

a Targets describe design requirements, not clinical treatment recommendations. Calendar support periods require indication- and device-specific validation.

Conventional treatment guidance also shows why benign disease should not automatically be equated with brief support. The 2026 European Society of Gastrointestinal Endoscopy guideline suggests six to twelve months of fully covered self-expandable metal stent (FCSEMS) treatment for chronic-pancreatitis-associated benign strictures. That duration describes an established treatment pathway, not a validated target for a degradable polymer. Disease phenotype and retreatment options must guide translation.^39^

## 5. Challenges and future perspectives

### 5.1 Platform maturity and remaining challenges

Despite the promising results from preclinical and early clinical studies, several challenges and controversies remain regarding the use of bioresorbable biliary stents. One major controversy is the optimal degradation timeline. The ideal degradation rate may also vary depending on the specific indication, with malignant strictures potentially requiring longer patency times than benign conditions. There is also debate regarding the optimal polymer composition and stent design. While PLA and PDO are the most commonly used materials, each has distinct advantages and disadvantages that may make them more suitable for specific applications. Table 7 distinguishes the advantages, limitations, developmental maturity, and unresolved challenges of each platform.^3^

**Table 7:** Bioresorbable platform advantages, limitations, and maturity^a^

Platform	Principal advantage	Principal disadvantage	Biliary maturity	Remaining challenge
PDO-based polymer stents	Flexible temporary framework; retrieval may be avoided	Fragmentation, migration, and inadequate support duration	Human feasibility/selected clinical series	Comparative recurrence and rescue outcomes; control fragmentation^14,33,36^
PLA and lactide-containing copolymers	Tunable composition and structural persistence	Persistence may outlast need; performance depends on processing	Predominantly laboratory/preclinical for the formulations discussed	Link molecular change and retained force to duct healing^11–13^
PGA devices	Rapid deterioration may suit a short support task	Early loss of mechanical integrity	Laboratory biliary phantom in the direct comparison	Demonstrate safe clearance and adequate support *in vivo*^5^
PDO/PLLA sheath-core	Longer retention in the tested phantom than single-material comparators	Interface failure and residual core require assessment	Biliary preclinical	Confirm reproducibility and human utility^5^
PDO/Mg hybrid	Potential reinforcement with degradable metal	Rapid degradation in the tested phantom	Laboratory biliary phantom	Control corrosion and preserve the polymer–metal interface^5^
Zinc-based metal systems	Strength, intrinsic attenuation, and gradual corrosion	Corrosion uniformity and local ion/product safety	Emerging preclinical	Demonstrate adequate support beyond the reported 3 weeks integrity and 8 weeks degradation findings in rabbits^9^
Functionalized fully degradable scaffold	Local therapy with eventual framework loss	Release and support may fail at different times	Laboratory/biliary animal	Establish complete-device benefit beyond drug-free controls and animal healing models^20,28^
Biomimetic or responsive systems	Potential tissue-directed or adaptive function	Complex manufacture, stability, and interacting biological effects	Conceptual/exploratory	Obtain direct bile-specific validation before human extrapolation^3^

a A biodegradable coating on a persistent metal stent is an adjacent coating platform, not evidence that the whole stent belongs in the fully bioresorbable category.

### 5.2 Technical challenges

Several technical challenges need to be addressed for successful clinical translation of bioresorbable biliary stents. One major challenge is achieving consistent and predictable degradation kinetics. The degradation rate of bioresorbable polymers can be affected by various factors including molecular weight, crystallinity, processing conditions, and the local environment. Bile composition, local pH, microbial deposits, and flow can modify degradation or drainage through different mechanisms, making it difficult to predict the exact timeline in clinical settings. Another challenge is maintaining sufficient mechanical strength throughout the healing period. Bulk-degrading polymers may lose mechanical strength before significant mass loss occurs, which can lead to stent collapse and obstruction. This requires careful design to ensure that the stent maintains patency until tissue healing is complete. Manufacturing challenges also exist, including the need for precise control over stent dimensions, coating uniformity, and drug loading. The production of bioresorbable stents requires specialized equipment and processes, which can increase costs compared to traditional stents.^10^

Preclinical testing should include delivery through the intended catheter or endoscope, expansion in a tortuous duct, coating damage during crimping, resistance to kinking, and fragmentation under combined bile exposure and loading. Healthy animal ducts alone may not reproduce fibrosis, malignant compression, or infection. Test outcomes should include tissue injury, microbial deposits, corrosion or degradation products, and drainage failure, with observation extending beyond visible framework loss.

Clinical protocols also need a rescue pathway. Premature loss of support, migration, cholangitis, retained fragments, and recurrent obstruction can require urgent drainage even when scheduled removal is unnecessary. The device must remain locatable and compatible with subsequent intervention. Comparative studies should stratify indication and access route, specify recurrence and reintervention endpoints, and measure outcomes after degradation, rather than ending follow-up when the stent disappears.

### 5.3 Regulatory and economic considerations

Regulatory approval represents a significant barrier to clinical translation of bioresorbable biliary stents because these devices combine degradable scaffolds, possible drug-device combination features, radiopaque modifiers, and sometimes multifunctional coatings. Biological evaluation should consider the final finished device, including sterilization, component interactions, manufacturing residuals, and changes during degradation. Additional development priorities include reproducible manufacturing, preservation of mechanical and release performance after sterilization, and coating stability during storage and delivery. Economic adoption will also depend on whether higher upfront costs are offset by fewer repeat procedures, fewer adverse events, and shorter overall care pathways.^40^

Drug-containing systems add requirements for reproducible loading and release and for characterization of tissue and systemic exposure. Radiopaque additives add another component whose fate must be understood. Manufacturing yield, shelf life, specialist delivery equipment, and the ability to manage complications affect economic adoption. A credible health-economic evaluation should use prospectively measured resources and patient outcomes, with sensitivity analyses for device cost, complication rates, and the number of avoided procedures.

### 5.4 Future directions

The first priority is a shared endpoint framework. Studies should measure mass loss, molecular-weight change, radial-force retention, fragmentation, release, and drainage in the same device under specified exposure conditions. Reporting these outcomes together would make apparently conflicting degradation times interpretable and permit comparison across laboratories.

The second priority is indication-matched comparative evaluation. Benign strictures, bile leaks, transplant anastomoses, and malignant obstruction should be analyzed separately. A coating should demonstrate benefit over an otherwise matched drug-free scaffold, followed by comparison with the appropriate current clinical pathway. Follow-up must include recurrence after framework loss, rescue procedures, patient-reported outcomes, and resource use.

Several future directions have been proposed to improve bioresorbable biliary stent technology, including stimuli-responsive drug delivery, patient-specific three-dimensional (3D) printing, advanced degradable composites, and sensor-enabled monitoring. Emerging biodegradable metallic biomaterials and metal-matrix composites may provide new combinations of strength, radiopacity, corrosion-controlled resorption, and manufacturability for next-generation biliary stents. Additive manufacturing, precision braiding, and multilayer coating routes could further enable patient-specific geometry and staged functional release, but these approaches remain exploratory until mechanical durability, sterilization compatibility, and clinical safety are demonstrated. Smart and shape-memory material systems may also support adaptive biliary stent designs that change configuration in response to temperature, pH, or other physiological cues. Such systems could improve deployment, conformability, or triggered release, but they should be framed as long-term research directions rather than near-term clinical solutions. Fig. 10 illustrates exploratory future directions for bioresorbable biliary stent technology, including responsive drug delivery, patient-specific manufacturing, multilayer release systems, and sensor-integrated monitoring. These concepts should be interpreted as research-stage opportunities that require validation under bile-specific mechanical, chemical, and microbial conditions before clinical use.^3,9^

**Fig. 10:**
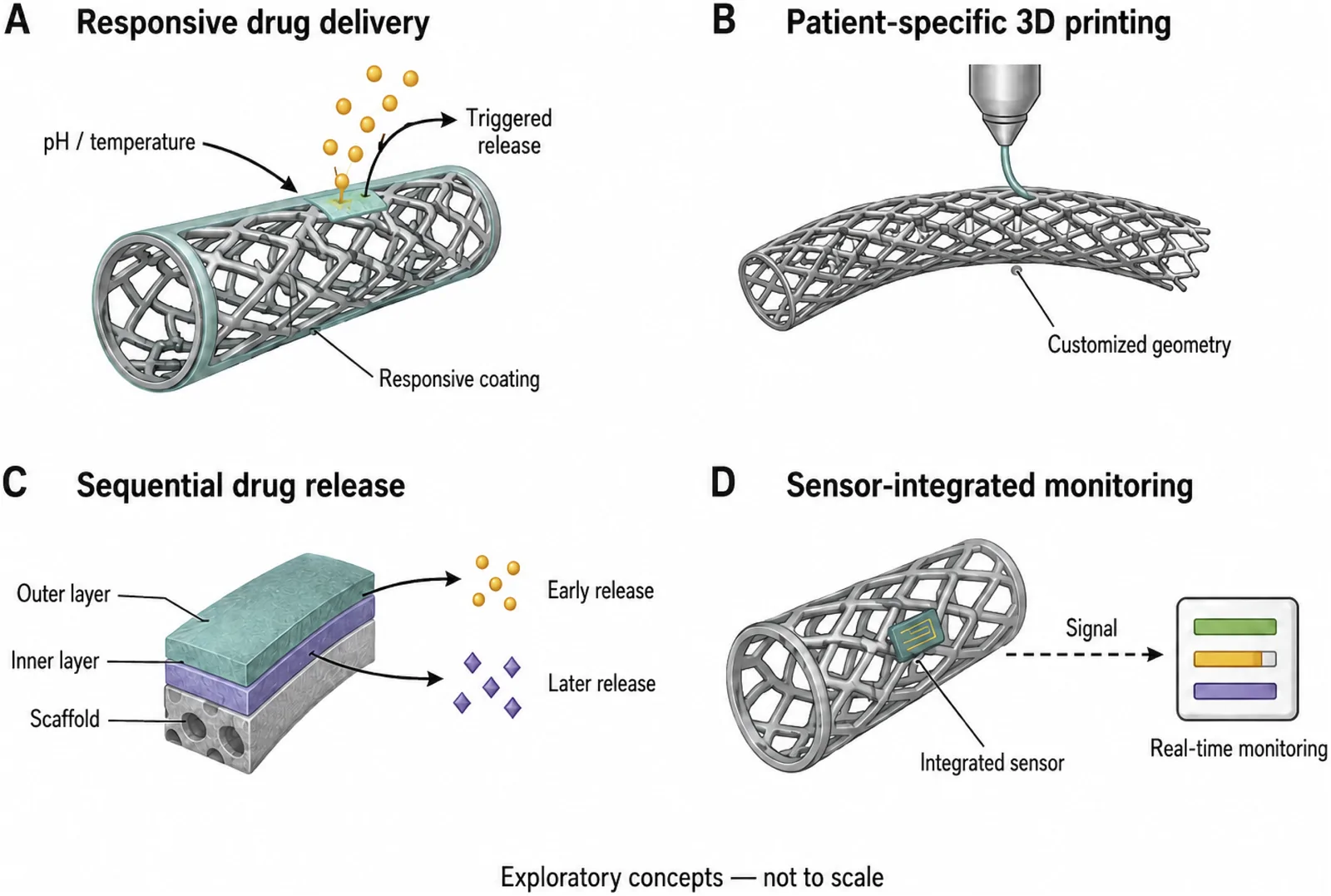
Future directions for bioresorbable biliary stent technology (A) smart stent with responsive drug delivery, (B) 3D-printed patient-specific stent, (C) multi-layered coating with sequential drug release, (D) biosensor-integrated stent for real-time monitoring. The panels are exploratory design concepts and are not drawn to scale.

## 6. Conclusion

Bioresorbable biliary stents represent an important but still maturing strategy for managing biliary strictures by integrating temporary scaffolding, controlled degradation, radiographic trackability, and local therapeutic function. The available evidence supports the rationale for indication-specific design, but mass loss, mechanical support, drug release, and biological clearance must be distinguished. Human evidence remains limited in comparative strength; metallic and multifunctional systems are predominantly preclinical. Future progress will depend on comparative studies that connect material behavior in real bile-rich environments with clinically meaningful outcomes, including recurrent obstruction, rescue intervention, and patient-reported outcomes after degradation.

## Author contributions

B. W. contributed to conceptualization, methodology, investigation, data curation, formal analysis, visualization, and writing – original draft. W. G. contributed to conceptualization, methodology, validation, writing – review & editing, supervision, and project administration. All authors have read and agreed to the published version of the manuscript.

## Conflicts of interest

There are no conflicts to declare.

## Data Availability

No new experimental data were generated or analyzed in this narrative review. The evidence discussed is available in the cited publications.
